# Evaluation of Large Language Models in Generating Physical Exercise Rehabilitation Programs for Musculoskeletal Disorders Across Multiple Clinical Scenarios

**DOI:** 10.3390/healthcare14152389

**Published:** 2026-08-04

**Authors:** Yu Fu, Hairui Li, Mingke You, Li Wang, Weizhi Liu, Kai Zhou, Lingcheng Wang, Xi Chen, Gang Chen

**Affiliations:** 1 Sports Medicine Center, West China Hospital, Sichuan University, Chengdu 610041, China; 2020141080306@stu.scu.edu.cn (Y.F.); 2024324020105@stu.scu.edu.cn (M.Y.); wangli1@stu.scu.edu.cn (L.W.); 2020141410222@stu.scu.edu.cn (W.L.); zhoukai_scu@scu.edu.cn (K.Z.); eric85264@hotmail.com (L.W.); 2 Department of Orthopedics and Orthopedic Research Institute, West China Hospital, Sichuan University, Chengdu 610041, China; 3 Department of Plastic Reconstructive and Aesthetic Surgery, West China Tianfu Hospital, Sichuan University, Chengdu 610041, China; pumchhrl@foxmail.com

**Keywords:** large language model, musculoskeletal disorder, physical exercise, rehabilitation program

## Abstract

**Highlights:**

**What are the main findings?**

- Four major large language models (LLMs) generated highly structured and consistent physical exercise rehabilitation programs for musculoskeletal disorders, achieving moderate-to-high quality with a mean DISCERN score of 55.60.
- Readability assessments across six validated indices revealed that the LLM-generated responses consistently required reading levels significantly higher than standard patient comprehension recommendations.

**What are the implications of the main findings?**

- While LLMs demonstrate substantial potential as auxiliary tools in digital orthopedics, their lack of supplemental resources and high reading difficulty hinder independent patient use.
- Professional physician oversight remains indispensable to bridge readability gaps, refine exercise descriptions, and ensure patient safety in AI-driven health consultations.

**Abstract:**

**Background:** Artificial intelligence and large language models (LLMs) are emerging as transformative technologies in medicine. However, their ability to develop physical exercise rehabilitation programs and provide insights into musculoskeletal (MSK) disorders remains underexplored. This study aimed to evaluate the quality and readability of LLM-generated responses to consultation questions addressing various stages of the clinical process encountered by patients with MSK disorders. **Methods:** This study recruited 50 patients with musculoskeletal disorders and extracted disease-related frequently asked questions from Google search. We developed three clinical scenario-based question types simulating real consultations, which were processed by four LLMs (GPT-3.5-turbo, GPT-4-turbo, GPT-4o, and Claude-3-haiku-20240307). Response quality was assessed by orthopedic specialists and therapists using the DISCERN instrument, and GPT-4o-assisted evaluation was used to extend the assessment after validation with expert ratings. Readability was systematically assessed via six validated indices. **Results:** Among the 1476 LLM-generated responses, the generated rehabilitation programs demonstrated consistent adherence to the specified query requirements. The DISCERN scores ranged from 26 (poor) to 68 (excellent), with a mean score of 55.60 ± 8.40. The intraclass correlation coefficient (0.68) indicated moderate interrater agreement, and Cronbach’s α showed good internal consistency. The readability scores across six indices indicated that most responses exceeded the recommended reading levels (*p* < 0.05). **Conclusions:** LLMs generated moderate-to-high-quality PE rehabilitation recommendations for patients with MSK disorders across simulated consultation scenarios. However, limited supporting materials and suboptimal readability may restrict their effectiveness. With physician oversight, improved readability, and enhanced supplementary resources, LLMs demonstrate considerable potential as supportive tools in orthopedics.

## 1. Introduction

Musculoskeletal (MSK) disorders pose a substantial challenge to patients, clinicians, researchers, and governments, ranking as the leading cause of disability worldwide and imposing a significant societal burden. Among individuals suffering from chronic pain, MSK disorders represent the most prevalent disease category, accounting for 70% to 80% of cases [1]. Prolonged occupational postures such as long-term sitting behind computer systems or sustained standing within industrial environments also constitute major contributors to MSK disorders in modern populations [2]. The most common types of MSK pain include low back pain (LBP), shoulder pain (SP), neck pain (NP), and pain associated with hip and knee osteoarthritis (OA) [3,4]. These MSK disorders represent a global health challenge, exerting considerable socioeconomic and personal burdens owing to their high prevalence and disability rates [5]. Physical exercise (PE) plays a pivotal role in both the prevention and rehabilitation of MSK disorders [6,7]. As recommended by clinical guidelines for LBP, PE, alone and in combination with educational interventions, plays a crucial role in both primary and secondary prevention and is considered the primary nonpharmacological treatment [8]. In managing pain associated with knee OA, the efficacy of PE is comparable to that of oral analgesics [9], and PE is widely utilized in pain management. PE has also been proven to be effective in alleviating pain and preventing further deterioration, as supported by robust evidence [10,11]. In summary, following established treatment guidelines, PE is acknowledged as an effective intervention for various MSK conditions and is typically administered by physical therapists.

As the global population grows and ages, the demand for PE has increased rapidly. However, this increasing demand cannot be adequately met due to shortages of physical therapists and healthcare resources [12] in many areas of the world, especially in low- and middle-income countries [13]. Effective exercise recommendations may vary owing to personal needs and individual characteristics such as age, contraindications, and body mass index (BMI) [14]. Nevertheless, these factors make it difficult to develop personalized PE rehabilitation programs for those suffering from MSK disorders. The scarcity of rehabilitation services and personal preferences often drive individuals to seek PE information online rather than consulting a healthcare professional. However, relevant literature suggests that much of the medical information found on the internet is inaccurate, misleading, and often contains unprofessional advice [15,16]. These factors significantly complicate the process for patients to obtain a personalized, professional, and high-quality PE rehabilitation plan for recovery from MSK disorders.

Generative artificial intelligence (AI), particularly large language models (LLMs), has recently shown increasing potential in clinical decision support, including medical consultation, triage assistance, and rehabilitation guidance [17,18]. Most existing studies investigating LLMs for MSK disorders have primarily focused on tasks such as information summarization, examination assistance, or isolated diagnostic support [19,20,21], demonstrating the potential of LLMs in improving clinical information processing and diagnosis while also highlighting limitations in clinical reasoning and patient-specific recommendations. Regarding PE program generation, previous work has successfully evaluated LLMs in specific postoperative rehabilitation protocols, such as anterior cruciate ligament reconstruction rehabilitation [22]. However, their ability to support rehabilitation decision-making for highly prevalent chronic and degenerative MSK conditions remains insufficiently explored. Moreover, existing evaluations remain fragmented and are largely restricted to isolated clinical problems or single-condition scenarios, leaving uncertainty regarding the applicability of LLMs in broader rehabilitation contexts [23,24,25]. This study focuses on PE rehabilitation programs, as they constitute core interventions endorsed by clinical guidelines for numerous MSK conditions, particularly chronic disorders such as low back pain and OA that require long-term, home-based management. This makes PE rehabilitation a suitable domain for evaluating the practical utility of LLM-generated clinical recommendations.

To address this gap, we designed a three-scenario clinical simulation framework that simulates the progressive stages of real-world patient–AI interaction in MSK care, ranging from unprompted patient queries (Scenario-1), to structured clinician-guided prompting (Scenario-2), and finally to complex, individualized, case-based reasoning (Scenario-3). This framework enables a systematic evaluation of LLM performance across different levels of clinical context complexity and prompt dependency, extending beyond single-protocol assessments to better reflect real-world rehabilitation decision-support scenarios. Therefore, the aim of this study was to evaluate the quality and readability of LLM-generated PE rehabilitation programs for patients with common MSK disorders, including OA and chronic pain conditions, across multiple clinically realistic interaction scenarios.

## 2. Methods

This study focused on five of the most prevalent MSK disorders worldwide: LBP, SP, NP, and hip and knee OA [26,27], with the global prevalence of LBP, NP, and OA reaching 59.52% in 2019 [28]. For each disorder, we designed requests on the basis of subtypes defined in relevant guidelines and various clinical scenarios to get more detailed exercise plans [5,8,10,29]. We used these standardized requests to ask four different LLMs, GPT-3.5-turbo (OpenAI, San Francisco, CA, USA), GPT-4-turbo (OpenAI, San Francisco, CA, USA), GPT-4o (OpenAI, San Francisco, CA, USA) and claude-3-haiku-20240307 (Anthropic, San Francisco, CA, USA), to provide PE rehabilitation programs for readability and quality assessment. These specific frozen versions were selected to ensure a consistent evaluation environment throughout the study and to avoid potential confounding introduced by continuous model updates during data collection and scoring. In accordance with the guidelines from the American College of Sports Medicine (ACSM), we adopted the FITT-VP principle as a framework for exercise plans, encompassing frequency, intensity, time, type, volume, and progression of exercise interventions [30,31]. We excluded referred pain from this study due to clinical safety considerations. The evaluation of referred pain requires physical and neurological examinations to rule out serious underlying conditions, which cannot be performed using text-based large language models. Therefore, the inclusion of such cases may introduce a risk of inappropriate interpretation and unsafe rehabilitation recommendations.

### 2.1. Clinical Scenario Simulation

The consultation questions of MSK patients were categorized according to content, volume, and the level of detail provided to the LLMs, with the aim of simulating potential clinical scenarios. We simulated three counseling situations: prediagnosis information gathering (Scenario-1), postdiagnosis clinical inquiries (Scenario-2), and postdiagnosis individual case analysis (Scenario-3).

#### 2.1.1. Scenario-1

**Figure 1:**
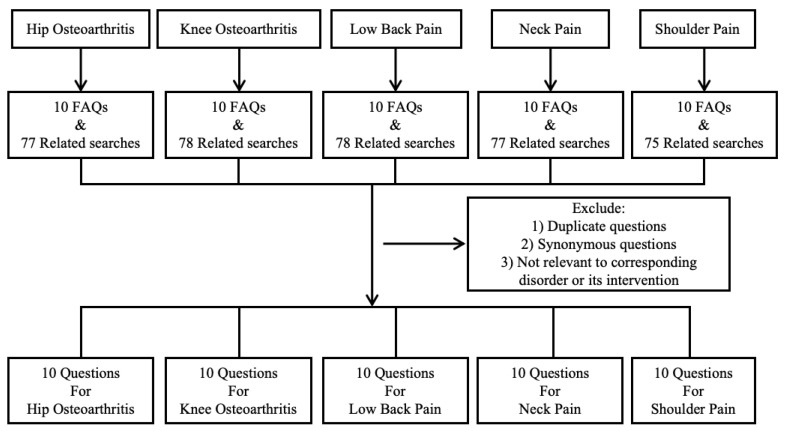
Selecting strategy using the frequently asked questions for the prediagnosis searching stage (Scenatio-1 stage).

In the prediagnosis searching stage (Scenario-1), we focused on the situation in which patients experienced early symptoms such as chronic pain but had not yet received a specific diagnosis. During this stage, patients often search for symptom-related information online. To emulate the questions that patients might ask, we used the most frequently asked questions (FAQs) associated with the five MSK disorders. The search strategy for collecting questions prior to exclusion in Scenario-1 is detailed in Figure S1. To minimize potential variability in Google search results caused by regional differences and personalized search algorithms, all searches were conducted from the same geographical location and network environment using a newly installed Google Chrome browser (Google LLC, Mountain View, CA, USA), Version 115.0.5790.3. The newly installed browser refers to a browser in which all site data, cached images, cookies, files, and browsing history had been cleared. No additional search filters or personalization settings were applied during the search process. Moreover, after searching for each MSK disorder type, we reinstalled the browser to eliminate any residual effects from previous searches. The inclusion criteria for the questions were: (1) they included any of the terms “low back pain,” “neck pain,” “shoulder pain,” “knee osteoarthritis,” or “hip osteoarthritis”; and (2) they mentioned “therapy,” “relief,” “exercises,” or “stretches.” Questions were excluded if they were duplicates, synonymous, or irrelevant to the corresponding disorder or its intervention (e.g., “How long should you lay on a heating pad for back pain?”). After selection, a total of 50 questions (10 questions without prompts per MSK disorder) were included, with an overview of the Scenario-1 question selection strategy presented in Figure 1. The selected basic questions for Scenario-1 provided to the LLMs are documented in Table S1, and the excluded questions are documented in Table S2.

#### 2.1.2. Scenario-2

In the postdiagnosis clinical consultation stage (Scenario-2), we conducted a study that focused on a scenario in which patients seek guidance from physical therapists after receiving a diagnosis of a specific disorder, subtype, or clinical stage. During this process, patients are typically aware of their diagnosis, and we simulated a scenario where doctors may assist them in developing rehabilitation programs that generally adhere to the FITT-VP principle [9]. In this scenario, LLMs were provided with diagnostic information and instructed to follow the FITT-VP framework to generate PE rehabilitation programs. Additionally, we asked LLMs to enumerate the objectives and rationales for each PE rehabilitation program to facilitate further quality evaluation. The only distinction in consultation queries was the specific diagnosis of the patients. At this stage, we excluded queries related to pain linked to specific pathologies, unique physical conditions, or those involving scheduled or recent surgeries. For knee and hip OA, the Kellgren–Lawrence grading scale was used for classification [32]; for LBP, we divided it into radicular and nonradicular subtypes [8], and for NP and SP, we utilized each condition’s common clinical presentations [10,29]. The consultation clues were structured as follows: “I was diagnosed as XXX, please according to the FITT-VP principle give me a PE rehabilitation plan and list the goal and reason of each stage.” where “XXX” represented the disease or disorder. We selected 23 basic queries provided to LLMs in Scenario-2, as shown in Table S3.

#### 2.1.3. Scenario-3

In the postdiagnosis individual case analysis stage (Scenario-3), we focused on the consultations of patients with clinical doctors to obtain personalized PE rehabilitation programs on the basis of specific diagnoses. During this process, doctors commonly formulate personalized PE rehabilitation programs tailored to the patients’ unique conditions, with patients’ information systematically recorded and collated in a standardized format. Considering that direct interaction between patients and LLMs is currently limited in clinical settings, the requests were entered by MSK health professionals. We adhered to a commonly used clinical format for designing PE rehabilitation programs (Figure S2), instructing LLMs to create treatment plans on the basis of detailed patient profiles and medical conditions. The questions in this scenario also specified adherence to the FITT-VP principle, including the objectives and rationales for each stage of the rehabilitation program. The basic consultation prompt of the questions was structured as follows: “According to the patient’s information, please generate a personalized detailed PE rehabilitation plan and follow the FITT-VP principle. Please list the reason and goal for each phase of the plan. After generating the plan, please check it. Here is the patient information you will use.”

**Figure 2:**
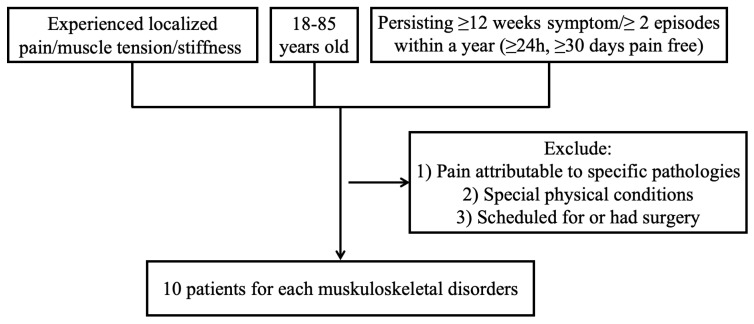
Inclusion and exclusion criteria for patient selection during the post-diagnosis individual case analysis phase (Scenario-3 stage).

In Scenario-3, an experienced clinician was responsible for patient recruitment, and patients were eligible if they: (1) experienced localized pain, muscle tension, or stiffness in the corresponding region; (2) were aged between 18 and 85 years; and (3) had symptoms persisting for more than 12 weeks or had experienced at least two episodes within a year, each lasting more than 24 h, with more than 30 days being pain-free. Exclusion criteria included pain attributable to specific pathologies such as fractures, ankylosing spondylitis, spondyloarthritis, infections, neoplasms, or metastasis, conditions such as pregnancy, as well as patients who were scheduled for or had undergone surgery. Figure 2 presents an overview of the patient selection strategy for Scenario-3. A total of 50 patients were included in this study, and a summary of the patients’ information provided to LLMs at this stage is available in Table S4. In this scenario, a total of 50 basic questions were provided to LLMs to generate PE rehabilitation programs. Ethical approval was obtained from the institutional review board (West China Hospital of Sichuan University Biomedical Research Ethics Committee) with official approval code 2023191, and all patients provided written informed consent.

### 2.2. Prompt Design

Studies have shown that prompt words can improve the readability of LLM-generated responses [33,34]. We categorized three prompt strategies for generating simplified LLM responses: no prompt, identity prompt, and Chain-of-Thought (CoT) prompt, the latter two of which have been proven to enhance LLM capabilities [35]. We applied these prompt words to the basic questions across all three scenarios described above. The design format for prompt words is provided in Table S5. After adding the prompts, we obtained a total of 150 questions for Scenario-1, 69 for Scenario-2 and 150 for Scenario-3.

### 2.3. Question Input

To ensure the independence of each LLM response, we entered each question into the LLMs through an application programming interface (API), with each question submitted on a new session without previous conversation history to avoid the influence from previous interactions or external websites. During the Scenario-1 and Scenario-2 phases, structured questions were directly input into the LLMs to obtain corresponding treatment plans. In Scenario-3, owing to the incorporation of patient data and the sensitivity of medical information, we followed a meticulous anonymization guideline (outlined in Figure S3) before inputting any clinical records to the LLMs. All LLM responses were documented in an Excel file for subsequent quality and readability analysis. Importantly, this study did not involve patient intervention, and all treatment recommendations or related content generated by the LLMs were not applied in clinical practice. The input process was conducted by an experienced doctor (Doctor-1) and a therapist (Therapist-1), with a second doctor (Doctor-2) independently checking the accuracy of inputs and the completeness of LLM responses.

### 2.4. Evaluation Procedures

#### 2.4.1. Quality Assessment

To evaluate the quality of rehabilitation programs generated by the LLMs, we established a human expert evaluation process and subsequently applied an LLM-assisted evaluation approach to extend the assessment to the remaining responses due to the substantial workload. The categories of the PE rehabilitation programs and their respective evaluation methods are detailed in Table S6.

For the human rating process, we invited three seasoned orthopedic doctors (Doctor-1, Doctor-2 and Doctor-3), each prescribing over 300 PE programs annually, and two therapists (Therapist-1 and Therapist-2), each performing more than 200 PE programs per year. The evaluators were fully blinded to group allocation, including model type and prompting strategy, and had no access to any patient-related or scenario-specific information during the assessment process. All five experts independently evaluated programs via the DISCERN tool, a validated and reliable instrument for assessing the quality of health information on treatment options [36]. The DISCERN criteria and scoring instructions were provided to all evaluators before assessment. The DISCERN instrument consists of 16 distinct questions (Table 1), with each question rated on a 5-point scale (1 = definitely no, 5 = definitely yes). The questions are organized into three sections: Questions 1 to 8 focus on the reliability of the content, Questions 9 to 15 examine the quality of treatment information, and Question 16 evaluates the overall quality of the treatment plan. The final DISCERN score of a program is the sum of all question scores, with higher scores indicating better quality and greater clinical relevance. Online health information on treatment choices is rated by the total DISCERN score, ranging from 16 to 80, and categorized as excellent (80 to 63), good (62 to 51), fair (50 to 39), poor (38 to 27), and very poor (26 to 16) [37].

In the LLM-assisted rating process, ChatGPT-4o was used as a supplementary assessment tool to evaluate the remaining LLM-generated responses after human expert validation. ChatGPT-4o has demonstrated potential in evaluating textual content quality in a previous study [38]. We provided ChatGPT-4o with the same instructions, content, and evaluation questions used in the human assessment. In this process, we used an API, instructing GPT-4o to follow the DISCERN tool to assign scores using a 1–5 scale and provide explanations for each score. The DISCERN questions used in both the human and LLM evaluations are detailed in Table S7.

To assess the concordance between LLM-assisted evaluations and human expert assessments, we randomly sampled the LLM’s evaluation scores and explanations and asked experts to review and rate them on a 1–5-point scale (1 = very poor quality and 5 = excellent quality). Additionally, we calculated the intraclass correlation coefficient (ICC) between the DISCERN scores of the five experts and those of GPT-4o. The specific strategy for random sampling and expert validation of the LLM evaluation scores is provided in Supporting Information S2.

To assess concordance among evaluators, we calculated the ICC of the DISCERN scores for each rehabilitation plan to evaluate interrater reliability while accounting for the possibility of chance agreement. An ICC value of 1 indicates perfect agreement, whereas an ICC value of 0 signifies agreement purely by chance. Agreement levels are categorized as follows: excellent (ICC > 0.90), good (0.75 < ICC ≤ 0.90), moderate (0.50 < ICC ≤ 0.75), and poor (ICC ≤ 0.50) [39]. To assess the internal consistency of the items, Cronbach’s alpha value was calculated, along with a 95% confidence interval (CI), based on the scores from 16 DISCERN questions evaluated by five experts. An alpha value > 0.70 indicates good reliability, whereas a value < 0.20 signifies poor reliability [40,41].

#### 2.4.2. Readability Evaluation

To prevent misinterpretations and confusion commonly encountered by patients due to obscure medical terminology, it is essential to evaluate the readability of generated rehabilitation programs and assess potential bias. To reduce human error, we used an API to evaluate the readability of LLM-generated responses according to six readability formulas (Table 2): Flesch–Kincaid Grade Level (FKGL), Flesch Reading Ease Score (FRES), Simplified Measure of Gobbledygook (SMOG), Gunning Fog (GF), Automated Readability Index (ARI), and Coleman–Liau Index (CLI). These formulas have been validated for assessing the readability of healthcare and patient information [42]. FKGL and FRES primarily assess average sentence length and syllables per word [43,44]. GF considers sentence length and the number of polysyllabic words containing three or more syllables [45]. SMOG evaluates polysyllabic words in three 10-sentence samples from the beginning, middle, and end of the text [46]. CLI and ARI are based on the number of letters and average sentence length, incorporating a correction factor for texts containing fewer than 30 sentences [47,48].

In accordance with guidelines from the US Department of Health and Human Services (USDHHS), healthcare information should be written at a level comprehensible to 11–12-year-olds, roughly equivalent to the sixth grade. In this study, materials were deemed easy to read if they had a readability score > 80 for FRES [49] or <6.9 for FKGL, SMOG, GF, CLI, and ARI. The USDHHS categorizes reading levels into three groups: easy (below 12 years old), average (12 to 15 years old), and difficult (above 15 years old). The scales for each readability metric used in this study are detailed in Table 3.

#### 2.4.3. Statistical Analysis

All statistical analyses were conducted using R software version 3.6.0. (R Foundation for Statistical Computing, Vienna, Austria) Means and standard deviations (SD) were calculated. Data normality and homogeneity of variance were evaluated using the Lilliefors-corrected Kolmogorov–Smirnov test and Levene’s test, respectively. Differences between groups were evaluated via the one-way analysis of variance (ANOVA) to compare means across multiple groups based on a single independent variable, with *p* < 0.05 deemed statistically significant. For large datasets, where formal tests often flag negligible deviations from strict normality, parametric ANOVA was maintained based on its established robustness under the Central Limit Theorem. Considering the hierarchical structure of the data, where multiple responses were generated for the same clinical questions, linear mixed-effects models were additionally performed as sensitivity analyses. LLM type, prompt strategy, and clinical scenario were treated as fixed effects, while question identity was included as a random effect.

## 3. Results

Upon receiving a rehabilitation query, LLMs generated a PE plan related to MSK disorders, with formats varying across scenarios. A total of 369 questions were submitted to four LLMs, generating 1476 responses. Of these, 40.65% (600/1476) pertained to Scenario-1, 18.70% (276/1476) to Scenario-2, and 40.65% (600/1476) to Scenario-3. In Scenario-1, rehabilitation programs generated by the LLMs were consistently structured into three segments: a title or basic introduction to the exercise, the exercise name, and a detailed description or instructions for each exercise, with or without safety precautions. Scenario-2 responses consisted of two or three parts: a brief introduction explaining the FITT-VP principle, a detailed framework of the rehabilitation plan, and, in some cases, a concluding section with safety notes. In this scenario, the detailed framework included multiple phases specifying the goals, frequency, intensity, time, type, volume, and progression in accordance with the FITT-VP principle. The Scenario-3 responses were more comprehensive, comprising five parts: a title, an explanation of the FITT-VP principle, a multiphase exercise plan structured similar to Scenario-2, general tips or recommendations, and a final check section to ensure accuracy and completeness.

All 1476 PE rehabilitation programs were evaluated using the DISCERN instrument. The mean, standard deviation, and range of DISCERN scores for 123 programs without prompts, generated by GPT-4, were assessed by three doctors, two therapists, and GPT-4o, as shown in Table 4.

In the human evaluation, the final mean ± SD score of these 123 plans was 50.48 ± 8.15. The ICC among the five experts was 0.68 (95% CI: 0.66–0.69), indicating moderate agreement among raters. Although Doctor-3 assigned relatively lower DISCERN scores compared with the other evaluators, this variability was considered when interpreting the inter-rater reliability and overall quality assessment results. We calculated the mean score of the five experts to represent the human score for each of these 123 responses and categorized them according to DISCERN criteria. The distribution of scores was as follows: very poor 0% (0/123), poor 6.51% (8/123), fair 38.22% (47/123), good 54.47% (67/123), and excellent 0.8% (1/123).

Prior to conducting intergroup comparisons, parametric test assumptions were validated using the full sample of 1476 observations. The Lilliefors test detected a statistically significant departure from perfect normality with *p* < 0.001. This outcome frequently emerges in analyses with large sample sizes and does not preclude parametric testing. Visual inspection of distribution curves revealed only mild skewness, supporting the application of ANOVA based on the Central Limit Theorem. Levene’s tests further verified homogeneity of variance for all primary outcome measures. For instance, the Levene test for core quality scores across disease subgroups returned F(4, 1471) = 0.75 with *p* = 0.56. All remaining measured variables produced *p* values greater than 0.05, confirming consistent variance across groups.

One-way ANOVA revealed a statistically significant difference among the scores assigned by the five experts (F (4, 611) = 49.12, *p* < 0.05). However, subsequent Tukey’s test indicated no significant difference between the scores of the two therapists and Doctor-2. The scores of Doctor-1 were relatively higher than those of other experts, whereas the scores of Doctor-3 were significantly lower. We also found a statistically significant difference in DISCERN scores across scenarios, with Scenario-1 scoring significantly lower than Scenario-2 and 3, and Scenario-2 scoring lower than Scenario-3. Conversely, no significant differences were observed in the average DISCERN scores across the five types of MSK disorders (*p* = 0.09).

To assess the concordance and feasibility of LLM-assisted evaluations, we randomly sampled 2180 DISCERN scores from a total of 23,616 scores (16 scores per LLM response) with a 95% confidence level and a 2% margin of error. One doctor (Doctor-1) and one therapist (Therapist-1) reviewed the LLM-provided scores along with their explanations, using a five-point Likert scale [50]. The mean ± SD ratings of the LLM-generated evaluation outputs were 4.80 ± 0.72 and 4.75 ± 0.75, respectively, suggesting that the explanations and scoring rationales provided by GPT-4o were generally considered appropriate by the reviewers. The ICC between those two experts was 0.90 (95% CI: 0.89–0.90), demonstrating good agreement between the two reviewers. We further assessed the concordance between GPT-4o-assisted DISCERN scores and human expert scores for responses generated by GPT-4 without prompts. The ICC was 0.62 (95% CI: 0.60–0.64), indicating moderate concordance with human expert assessment.

**Figure 3:**
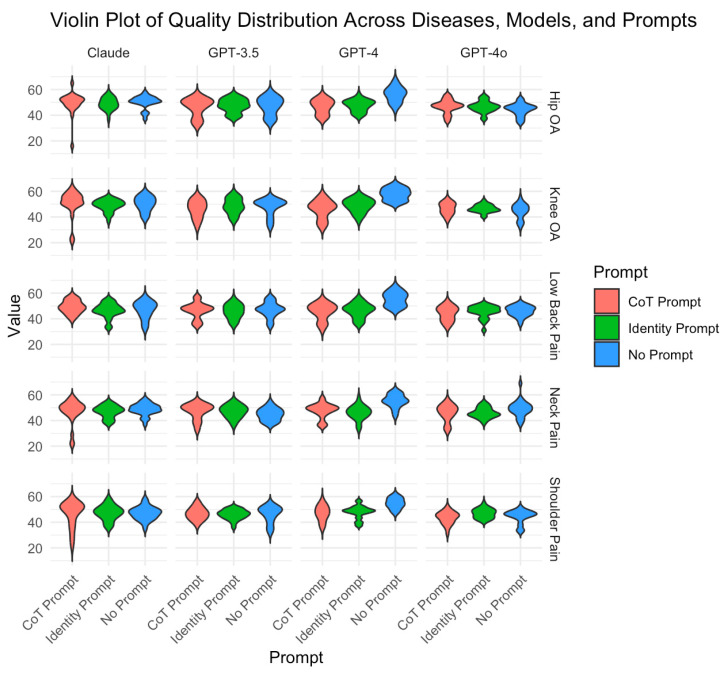
Violin plot of DISCERN scores across MSK disorders, models, and prompts. DISCERN scores of all 1476 LLM-generated responses across MSK disorders, LLMs, and prompts. Different colors denote the various prompt types provided to the LLMs.

In the GPT-4o-assisted evaluations, we calculated the mean, SD, and range for all programs, grouping them by prompt type and the model used, as documented in Table S8. After the total DISCERN scores across 1476 responses assessed through GPT-4o-assisted evaluation were calculated, the distributions were as follows: very poor 0.47% (7/1476), poor 9.15% (135/1476), fair 57.59% (850/1476), good 31.64% (467/1476), and excellent 1.15% (17/1476). A violin plot illustrating the distribution of DISCERN scores for all programs is provided in Figure 3. One-way ANOVA revealed statistically significant differences in mean DISCERN scores when grouped by MSK disorder type (F(4, 1471) = 3.23, *p* = 0.01, partial η^2^ = 0.009), scenario (F(2, 1473) = 221.1, *p* < 0.05, partial η^2^ = 0.231), model (F(3, 1472) = 31.64, *p* < 0.05, partial η^2^ = 0.061), and prompt type (F(2, 1473) = 20.58, *p* < 0.05, partial η^2^ = 0.027). Pairwise comparisons revealed that for model architectures, GPT-4 significantly outperformed Claude (mean difference = 1.64, *p* < 0.001) and GPT-3.5 (mean difference = 3.25, *p* < 0.001), while GPT-4o consistently demonstrated lower scores than GPT-4 (mean difference = −4.11, *p* < 0.001). Regarding prompting strategies, employing “No Prompt” resulted in significantly better quality scores than utilizing a “Chain of Thought (CoT) Prompt” (mean difference = 2.38, *p* < 0.001) or an “Identity Prompt” (mean difference = 2.06, *p* < 0.001). For clinical conditions, significant differences in human-evaluated quality were sparse, isolated primarily to Shoulder Pain vs. Knee OA (mean difference = −1.56, *p* = 0.03) and Low Back Pain vs. Knee OA (mean difference = −1.49, *p* = 0.04). The results of other subsequent Tukey’s tests are presented in Table S9.

Sensitivity analyses using linear mixed-effects models yielded consistent findings, with significant effects observed for LLM type, prompt strategy, and clinical scenario (Table S10).

When assessing the internal consistency of experts’ evaluations of responses generated by GPT-4 without prompts, the Cronbach’s α value based on the original 16-item DISCERN instrument was 0.83. During exploratory item-level analysis, Q4 and Q5 showed negative correlations with the principal component, whereas Q12 demonstrated limited variability across responses. After reviewing the content of these items, Q4 and Q5 mainly assessed the availability and evaluation of information sources, while Q12 focused on alternative treatment options, which were less applicable to AI-generated PE rehabilitation programs. Therefore, Q4, Q5, and Q12 were excluded only for this subgroup reliability analysis, and the Cronbach’s α increased slightly to 0.84, indicating good internal consistency. The original 16-item DISCERN scoring system was retained for all primary quality assessments.

The Cronbach’s α value for the GPT-4o-assisted DISCERN scores was 0.80, with all items positively correlated with the principal component.

**Figure 4:**
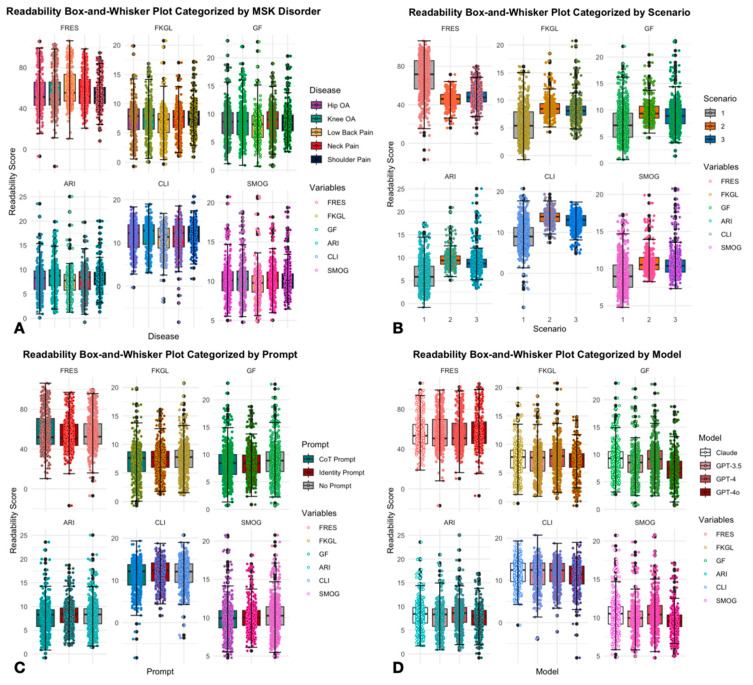
Box-and-whisker plots of readability scores. Readability scores for all 1476 rehabilitation programs: (**A**) box-and-whisker plots of readability scores categorized by MSK disorder. (**B**) Box-and-whisker plots of readability scores categorized by scenario. (**C**) Box-and-whisker plots of readability scores categorized by prompt type. (**D**) Box-and-whisker plots of readability scores categorized by LLM.

**Figure 5:**
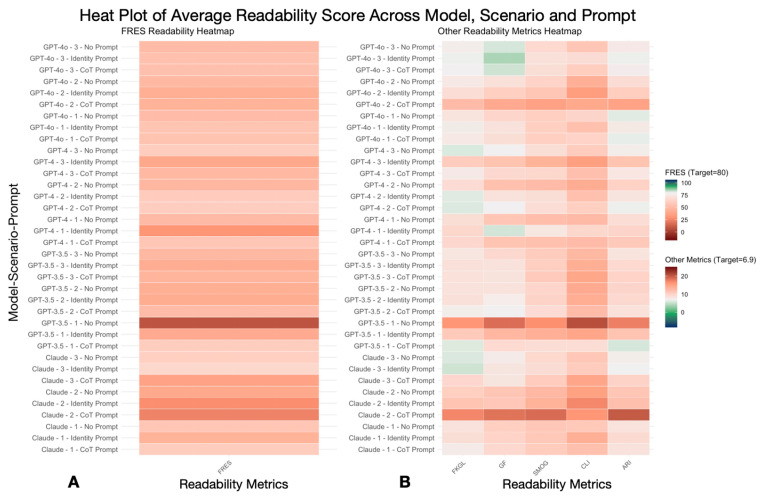
Heat plot of average readability score across model, scenario and prompt. Readability scores are displayed by model, scenario, and prompt, with colors indicating readability levels: white denotes the recommended readability threshold (80 for FRES and 6.9 for other metrics), green signifies readability exceeding the recommended level, and red indicates poor readability. (**A**) Readability scores of FRES. (**B**) Readability scores of FKGL, GF, SMOG, CLI, and ARI.

All 1476 rehabilitation programs underwent readability testing. The mean (SE) scores across the six readability tools for all programs were as follows: FRES: 56.54 (17.86), FKGL: 7.30 (2.82), GF: 8.52 (3.00), SMOG: 10.11 (2.16), CLI: 11.47 (3.33), ARI: 8.01 (3.03). Most of the six readability assessment metrics reported statistically significant results compared to the recommended readability level scores (*p* < 0.05), indicating that the readability of the three-scenario programs falls significantly below the “easy-to-read material” standards outlined in the “Method” section (>80 for FRES and >6 for other indices). Subgroup analysis sorted the readability scores by “Scenario”, “MSK disorder”, “Prompt” and “Model.” The distribution of readability scores of these groups is illustrated in box-and-whisker plots (Figure 4). Results from the one-way ANOVA and subsequent Tukey’s tests of readability indicators revealed that Scenario-1 yielded better readability outcomes than Scenarios 2 and 3 for all six evaluation metrics, accompanied by reduced FKGL values and elevated FRES values (*p* < 0.001). Comparisons between different models showed that texts generated by GPT-4o had significantly lower reading grade levels relative to GPT-4. This difference was supported by the FKGL (mean difference = −0.72, *p* < 0.001), GF (mean difference = −1.72, *p* < 0.001), and SMOG (mean difference = −0.95, *p* < 0.001) indices. Among all MSK disorders, materials developed for low back pain exhibited greater reading difficulty than those for hip osteoarthritis and knee osteoarthritis across multiple readability metrics (*p* < 0.05). The results of other subsequent Tukey’s tests are provided in Table S11. The average scores for each model, prompt, and scenario are presented in a heat map (Figure 5), providing a clearer visualization of the distribution of readability scores, and highlighting which readability scores exceeded the “easy to read” threshold.

## 4. Discussion

To our knowledge, this is the first study to evaluate LLMs using a multi-scenario clinical workflow simulation framework for generating PE rehabilitation programs in MSK rehabilitation. Our study simulated three clinically realistic scenarios for five common MSK disorders to examine the applicability and generalizability of LLMs in routine rehabilitation practice. We designed three types of prompts to examine the impact of questioning methods on the readability of responses. Ultimately, we received 1476 rehabilitation program responses by providing instructions to GPT-3.5, GPT-4, GPT-4o, and Claude-3 Haiku. The DISCERN tool was used to evaluate the quality of the generated plans and six readability tools were used to test the readability. Three professional doctors and two experienced therapists independently assessed the quality of 123 programs generated by GPT-4 without adding identity or CoT prompts. Due to the substantial workload associated with evaluating the remaining responses, GPT-4o was subsequently used as a supplementary assessment tool after human validation. The concordance between GPT-4o-assisted evaluations and human expert assessments was examined through ICC analysis and additional expert validation of randomly sampled scores. Results indicated moderate agreement between GPT-4o-assisted evaluations and human expert assessments, suggesting that GPT-4o may facilitate large-scale assessment but should not replace professional evaluation.

Over the past decade, the number of internet users and visits to health-related websites have steadily increased, making the internet a critical source of health information for the public [51]. However, internet health information has numerous issues such as inaccuracies, insufficient detail, a lack of personalization, misleading content, and even occurrences of errors. Several studies that utilized DISCERN as an assessment tool have indicated that the quality of health information provided by internet sites is relatively poor [37,52,53]. A greater availability of online platforms capable of delivering accurate, personalized, and high-quality health information is essential. Such platforms would offer patients more choices and better support in managing their health issues. AI chatbots have demonstrated potential in clinical consultation scenarios and may help address the aforementioned issue; however, further testing is required to assess the quality and readability of their outputs. Consequently, we employed six readability testing tools and the DISCERN instrument to evaluate the responses we obtained.

Our study demonstrates that LLMs consistently generated moderate-to-good-quality PE programs for all MSK disorder stages, although readability remained challenging. Moreover, LLMs can generate more individualized PE rehabilitation programs based on patient-specific information provided in the prompts. However, LLMs generally did not provide the source of supporting information and lacked descriptions of what would happen if no treatment were used. These findings suggest that LLMs may serve as a potential auxiliary tool for providing preliminary rehabilitation-related information and supporting clinicians in the development of PE programs. However, their outputs should not be considered a substitute for professional clinical evaluation. Rather than serving as a simple benchmark comparison of LLMs, our study provides a structured evaluation of how model performance changes across progressively more realistic clinical consultation scenarios and prompting strategies in MSK rehabilitation. By integrating model comparison, scenario simulation, and prompt engineering analysis within a unified experimental design, our findings provide a more clinically realistic assessment of LLM behavior than single-turn question-answering benchmarks commonly reported in the literature [54]. Collectively, these findings suggest that LLMs’ performance in MSK rehabilitation is highly dependent on the interaction between clinical scenario complexity and prompting strategy, highlighting the importance of workflow-aware evaluation rather than single-turn benchmarking.

The moderate agreement among human evaluators warrants further consideration. Although all evaluators independently assessed the generated programs according to the DISCERN criteria, variability existed among individual raters, particularly for Doctor-3, who assigned relatively lower scores than the other evaluators. This difference may reflect variations in clinical judgment and evaluation standards rather than inadequate assessment reliability. Experienced clinicians may apply different thresholds when assessing the completeness, safety, and clinical applicability of AI-generated recommendations. In particular, more senior experts may adopt stricter criteria when evaluating whether generated rehabilitation programs meet real-world clinical expectations. Therefore, variability among evaluators may represent the inherent complexity of assessing AI-generated clinical recommendations, highlighting the importance of multi-expert evaluations rather than reliance on a single reviewer.

In our tests, when requiring LLMs to outline FITT-VP principles, set objectives, perform step-by-step planning, and explain rationales, LLMs demonstrated commendable compliance by accurately following our instructions and independently listing the relevant sections. Regardless of the type of LLM model, the type of prompt used, the type of MSK disorder or the scenario, LLMs consistently generated clinically relevant rehabilitation information, without significant errors. These findings suggest that although minor performance differences existed across disease categories, LLMs consistently generated clinically acceptable rehabilitation programs regardless of the specific MSK condition. Additionally, we observed that as the scenario and prompt changed, the quality of the generated results improved, likely due to the varying requirements provided to LLMs in each scenario. Scenario-2, compared to Scenario-1, included two additional requirements: adherence to the FITT-VP principles and explaination of the goals and rationale of the plan. Scenario-3 built upon this by adding anonymized patient information, which provided LLMs with more details, significantly enhancing the quality of the generated rehabilitation programs. Additionally, in Scenario-3, LLMs considered the chief medical problem, exercise preferences, available equipment, exercise environment, and other personal patient information to generate comprehensive, individualized, and high-quality PE rehabilitation programs. This indicates that the format, content, and quality of LLMs’ output depend on the given instructions. Without specific directives, LLMs struggled to provide additional information on that topic. The outputs in Scenario-1 and Scenario-2 align well with our intended purposes, offering relatively high readability, especially in response to the simpler questions posed at Scenario-1, where LLMs provided concise answers. The setup of Scenario-3 met our expectations as well, with higher quality but lower readability. During this phase, we observed an abundance of medical terminology within the treatment plans, which may cause reading challenges for those without professional medical knowledge, yet poses no obstacle for clinicians. This demonstrates that LLMs are indeed capable of tailoring their responses based on the object of the conversation, underscoring its potential application in helping patients access health information and providing recommendations to doctors. Significant differences were observed between LLMs and prompt types. The DISCERN scores, ranked by model from highest to lowest, were as follows: GPT-4, Claude-3 Haiku, GPT-3.5, and GPT-4o. Upon reviewing responses, we found that some programs generated by GPT-4o focused strictly on the given requirements but lacked elements like a clear title or safety tips. This suggests that, while LLMs have improved in following instructions to produce more specific results, they may still lack comprehensive content beyond the direct prompts.

Additionally, responses generated without prompts exhibited higher quality compared to those with identity or CoT prompts. We reviewed the responses and found that identity and CoT prompts tended to guide LLMs towards providing simple, clear exercise tips, but often lacked logical flow or emphasis, which may have contributed to the observed reduction in quality. Two distinct mechanisms may explain this counterintuitive finding. One possible explanation for the poorer performance of identity prompts is clinical commonsense omission. When an LLM is instructed to adopt the persona of a clinical expert, it may emulate the way doctors communicate with patients in routine clinical practice, where basic medical information is often omitted because it is assumed to be common knowledge. Consequently, the generated responses may be less comprehensive and fail to satisfy the detailed criteria required by the DISCERN instrument. In contrast, without explicit prompting, LLMs may approach the task as a general information generation task, producing more comprehensive rehabilitation recommendations that better align with DISCERN evaluation criteria. Another possible explanation is that CoT prompting may shift the model’s focus toward explicit reasoning rather than comprehensive rehabilitation guidance. When generating lengthy clinical recommendations, this emphasis on intermediate reasoning may reduce the space available for practical exercise guidance. Consequently, the final recommendations may become more concise while providing less comprehensive clinical detail, thereby lowering DISCERN scores. These observations provide important guidance for clinical prompt design. Simple role playing or open reasoning prompts may not be optimal for generating patient-oriented rehabilitation materials and may reduce the completeness of the generated recommendations. Future clinical applications should consider more structured prompting strategies, such as few-shot prompts [55] based on established clinical scales or leverage Retrieval-Augmented Generation (RAG) systems [56] to improve the completeness, consistency, and safety of generated rehabilitation recommendations.

It should be noted that, in the DISERN evaluation process, questions Q4, Q5, and Q12 scored particularly low. Q4 and Q5 pertain to the assessment of information sources for the generated results, while Q12 addresses the description of the consequences of lack of treatment. A reliable information source is important for a high-quality rehabilitation program [57]. Additionally, during the selection process for treatment options, if no alternatives are available, it is vital for patients to understand the potential outcomes clearly. This understanding plays a significant role in safeguarding patients’ health and maintaining a positive doctor-patient relationship [58,59]. LLMs showed limitations in the above two aspects, likely due to the absence of direct instructions. However, we also observed that for the third result of Scenario-1 regarding Knee OA generated by GPT-4, four experts awarded a score of 5 under the Q4 scoring item, while one doctor rated it a 3. Our analysis of the LLM’s response revealed that, although it did not provide an exercise video for Knee OA as requested, it did offer two websites as sources of information, which we verified to be effective. This further corroborates the characteristic of LLMs discussed previously, where LLMs’ outputs are highly contingent upon the instructions received. This insight prompts us to take a more comprehensive approach when providing commands to LLMs, considering all facets of a rehabilitation treatment plan, including requiring LLMs to furnish supporting information and diverse treatment options, thereby enabling LLMs to generate high-quality results. However, we recognized that the general public’s lack of medical knowledge can complicate their ability to provide comprehensive and precise instructions to LLMs, thereby impacting the quality of the generated results and posing potential risks. This represents a significant challenge for LLMs in delivering medical services and information to the broader public and acts as a barrier to its widespread adoption in healthcare. Encouragingly, with advancements in multimodal LLMs, a wider range of information formats such as files, text, and even images, can be utilized in generating healthcare information, potentially addressing this issue [60,61].

In the readability test, the readability of the rehabilitation programs generated by LLMs was deemed fairly difficult, consistent with findings from earlier studies [62]. Compared to a survey in another study [54], the FRES for results generated by LLMs in this study was lower than that from Google, while the FKGL was higher, suggesting that without specific instructions, the readability of treatment plans produced by LLMs is inferior to that of results from Google. Studies have shown that LLMs can simplify health information and make it more readable [63]. Unlike static websites, LLMs can tailor simplified content to match the user’s comprehension level. However, this simplification process may lead to misunderstandings [64]. Medical terminology is obscure for non-medical professionals, indiscriminate simplification of medical text may lead to further misunderstandings or even alter its original meaning and confuse patients [65]. Several studies showed that LLMs may fabricate data or even provide incorrect information, which is unacceptable in clinical practice [66]. We designed three prompt types to examine the effect of prompts on the readability of the responses. However, quality assessments also indicated a decline in quality for responses generated using CoT and identity prompts. It should be emphasized that conventional readability indices mainly assess linguistic complexity rather than actual patient comprehension, as they do not fully capture factors such as health literacy and understanding of medical concepts. Therefore, future studies incorporating patient-centered comprehension assessments are needed to further evaluate the accessibility of LLM-generated rehabilitation recommendations.

Our findings demonstrate that while prompts can improve the readability of LLM responses, they may also reduce overall quality. This suggests that future optimizations are needed for LLMs to generate rehabilitation programs for MSK disorders that are both highly readable and of superior quality. Experienced orthopedic doctors could use LLMs as auxiliary tools to support the development and explanation of PE rehabilitation plans, while maintaining professional oversight and clinical judgment.

### Limitations

This study possesses several limitations. Although five experienced professional raters participated in the scoring process, they did not evaluate all LLM-generated responses, and GPT-4o was used as a supplementary tool for large-scale assessment. Although we evaluated the concordance between GPT-4o-assisted evaluations and human expert ratings, AI-based assessment should not be considered equivalent to professional clinical judgment. Future studies involving larger-scale human evaluations are warranted to further validate the quality and clinical applicability of LLM-generated rehabilitation recommendations. This study only focused on LBP, NP, SP, hip and knee OA, further research is needed to examine other MSK conditions. Considering patients’ accessibility to popular websites and their typical search preferences, we used Google FAQs to simulate potential patient inquiries about MSK disorders in Scenario-1, rather than relying on standardized questions and answers from authoritative sources [67]. But this approach caused the questions to vary based on search engine queries, ranking mechanisms, and location. Our structured questions may have led to homogeneity in the generated outcomes and potentially made the generated outputs appear more homogeneous. Although multiple validated readability indices were used in this study, these metrics primarily capture surface-level linguistic characteristics and cannot fully reflect conceptual complexity, health literacy, or actual patient comprehension. Future studies should incorporate direct patient evaluations or comprehension tests to better assess the accessibility of LLM-generated rehabilitation information.

Additionally, the questions in Scenarios 2 and 3 appear unlikely to represent queries that patients would initiate on their own. Future research could incorporate a broader range of question formats to enrich LLMs’ responses, better simulating the real-world inquiries patients encounter during their diagnosis and treatment process. In Scenario-2, the simulated scenarios might not fully reflect the challenges patients face in actual conditions, as our research positioned doctors as intermediaries, without direct questioning from patients to LLMs. Another limitation was encountered at Scenario-3, due to the relatively small size of the patient cohort. While this may be deemed acceptable for the initial exploratory phase, more extensive studies are imperative to ensure the findings’ applicability on a wider scale. We created a new blank page and restarted the conversation before providing the questions, this could potentially compromise the completeness of patient history collection by LLMs, thus affecting their output. We focused on English responses from the LLMs, which limits the external validity and generalizability of our findings to non-English-speaking populations where MSK disorders are highly prevalent. Future research should validate these models across other widely spoken languages, particularly Spanish and Portuguese, to evaluate how multilingual processing affects the clinical quality and readability of LLM-generated rehabilitation programs. Additionally, our study focused on evaluating responses from four specific LLMs to assess the effectiveness of current models. However, with the rapid advancement of AI, newly released LLM versions after our data collection may yield different results and potentially enhance the outcomes. This study primarily evaluated the quality and readability of LLM-generated rehabilitation programs using the DISCERN instrument which is a validated tool for assessing health information quality. However, the DISCERN instrument does not directly evaluate clinical accuracy, contraindications, patient safety, or the potential risk of inappropriate exercise recommendations. Therefore, the results should be interpreted as an assessment of informational quality rather than evidence that LLM-generated programs are clinically safe for direct patient implementation. Future studies should incorporate clinical accuracy assessment, safety evaluation, and outcome-based validation involving healthcare professionals and patients. Another limitation involves the post hoc exclusion of DISCERN items Q4, Q5, and Q12 during the subgroup reliability analysis of GPT-4 responses without prompts. Because the model outputs in this condition rarely included references or alternative treatment options, these items consistently received the lowest possible scores, resulting in a pronounced floor effect. Although excluding these items only marginally increased Cronbach’s alpha from 0.83 to 0.84, and sensitivity analysis confirmed that this adjustment did not alter the study’s primary conclusions, this modification represents a deviation from the original validated DISCERN instrument. Therefore, the resulting reliability estimates should be interpreted with appropriate caution in this specific subgroup analysis.

Despite these challenges, this study provides valuable insights into LLMs’ potential capability for generating PE rehabilitation programs for MSK disorders and offering healthcare information. LLMs may hold promise as an auxiliary consultation tool in orthopedics and might offer valuable suggestions during the creation of PE rehabilitation programs by clinicians. Although LLMs demonstrated commendable performance in this study, it is imperative to emphasize that they cannot replicate the extensive experience and judgment of clinical doctors, necessitating cautious use. Taken together, our findings suggest that evaluating LLMs within clinically realistic consultation workflows provides more actionable evidence for future rehabilitation applications than conventional single-turn benchmark evaluations.

## 5. Conclusions

In this study, we assessed the quality and readability of PE rehabilitation programs for five MSK disorders, generated by four evaluated LLMs across three simulated scenarios and three prompt types. The findings consistently demonstrated moderate-to-high-quality outputs across different clinical scenarios, MSK disorders, model types, and prompts, although some readability challenges were observed. Our results suggest that LLMs can follow specific instructions, showing their potential to generate clinically relevant responses for patients with MSK disorders. However, without explicit guidance, LLMs tend to lack supplementary information, highlighting the need for expertise and comprehensive input to maximize their utility.

As AI-driven LLMs continue to evolve, they hold promise for improving access to health information and providing potential support to clinicians in developing PE rehabilitation programs. Enhancing the accuracy and professionalism of AI-driven LLMs is essential for their broader future application. Importantly, AI cannot replace clinical practitioners, whose rich experience and professional expertise remain irreplaceable. Professional oversight and evaluation by medical professionals remain essential to ensure the safe and effective use of LLMs in diverse clinical contexts.

## Figures and Tables

**Table 1:** DISCERN questions.

ID	DISCERN Question
Q1	Are the aims clear?
Q2	Does it achieve its aims?
Q3	Is it relevant?
Q4	Is it clear what sources of information were used to compile the publication (other than the author or producer)?
Q5	Is it clear when the information used or reported in the publication was produced?
Q6	Is it balanced and unbiased?
Q7	Does it provide details of additional sources of support and information?
Q8	Does it refer to areas of uncertainty?
Q9	Does it describe how each treatment works?
Q10	Does it describe the benefits of each treatment?
Q11	Does it describe the risks of each treatment?
Q12	Does it describe what would happen if no treatment is used?
Q13	Does it describe how the treatment choices affect overall quality of life?
Q14	Is it clear that there may be more than one possible treatment choice?
Q15	Does it provide support for shared decision-making?
Q16	Based on the answers to all of the above questions, rate the overall quality of the publication as a source of information about treatment choices

**Table 2:** Calculating formula for each readability test tools.

Tool Name	Calculating Formula
FKGL	$FKGL\;Score=0.39\left(\frac{total\;words}{total\;sentences}\right)+11.8\left(\frac{total\;syllables}{total\;words}\right)-15.59$
FRES	$FRES\;Score=206.835-1.015\left(\frac{total\;words}{total\;sentences}\right)-84.6\left(\frac{total\;syllables}{total\;words}\right)$
SMOG	$SMOG\;Score=1.043\sqrt{\left[\left(total\;polysyllabic\;words\right)\times \left(\frac{30}{total\;sentences}\right)+3.1291\right]}$
GF	$GF\;Score=0.4[\left(\frac{total\;words}{total\;sentences}\right)+100(\frac{total\;polysyllabic\;words}{total\;words})]$
ARI	$ARI\;Score=4.71\left(\frac{total\;characters}{total\;words}\right)+0.5\left(\frac{total\;words}{total\;sentences}\right)-21.43$
CLI	$CLI\;Score=5.88\left(\frac{total\;characters}{total\;words}\right)-29.6\left(\frac{total\;sentences}{total\;words}\right)-15.8$

**Table 3:** Scale for six readability test tools.

FRES Readability Scale Score	FKGL, SMOF, CLI, GF or ARI Readability Scale Score	Age	USDHHS Reading Level
90–100	0–1	6–7 yr old	Very Easy
	2–3	7–8 yr old	
	3–4	8–9 yr old	
	4–5	9–10 yr old	
	5–6	10–11 yr old	
80–89 *	6–7 *	11–12 yr old *	Easy *
70–79	7–8	12–13 yr old	Fairly Easy
60–69	8–9	13–14 yr old	Average
	9–10	14–15 yr old	
50–59	10–11	15–16 yr old	Fairly Difficult
	11–12	16–17 yr old	
	12–13	17–18 yr old	
30–49	13–17	18–22 yr old	Difficult
0–29	17+	22+ yr old	Very difficult

* Recommended reading level.

**Table 4:** Summary statistics for DISCERN scores of GPT-4 generated programs (without prompts).

Rater Identity	Mean	Standard Deviation	Range
Doctor-1	55.60	8.4	34 to 68
Doctor-2	51.95	9.62	33 to 67
Doctor-3	40.89	6.88	26 to 56
Therapist-1	52.87	8.99	34 to 68
Therapist-2	51.19	10.23	30 to 67
GPT-4o	55.82	5.16	43 to 67

## Data Availability

The datasets used or analyzed during this study are available from the corresponding author upon reasonable request.

## Supplementary Materials

The following supporting information can be downloaded at: https://www.mdpi.com/article/10.3390/healthcare14152389/s1, Figure S1: Search strategy for acquiring questions prior to exclusion in Scenario-1 stage; Figure S2: Format of patient information of Scenario-3 questions; Figure S3: Anonymization strategy in Scenario-3 stage; Table S1: Questions provided to LLMs in Scenario-1 stage; Table S2: Excluded questions in Scenario-1 stage; Table S3: Basic questions provided to LLMs in Scenario-2 stage; Table S4: Summary of patients’ information in Scenario-3 stage; Table S5: Example prompt types; Table S6: LLMs’ generated results categories with their evaluation strategy; Table S7: Questions used to conduct human and LLMs evaluation; Table S8: Tukey’s Test Results of Readability Scores; Table S9: Tukey’s Test Results of DISCERN Scores; Table S10: Linear mixed-effects model analysis of LLM-generated response scores; Table S11: Tukey’s Test Results of Readability Scores; Table S12: Formula for Calculating the Size of Random Sampling and Z-scores Distribution; Table S13: Tips for experts’ validation of LLM’s evaluation.

## Abbreviations

The following abbreviations are used in this manuscript:

LLM	Large Language Model
OA	Osteoarthritis
LBP	Low Back Pain
SP	Shoulder Pain
NP	Neck Pain
MSK	Musculoskeletal
PE	Physical Exercise
GPT-4	Generative Pre-trained Transformer Version 4
BMI	Body Mass Index
AI	Artificial Intelligence
ACSM	American College of Sports Medicine
FAQs	Frequently Asked Questions
CI	Confidence Interval
FKGL	Flesch–Kincaid Grade Level
FRES	Flesch Reading Ease Score
SMOG	Simplified Measure of Gobbledegook
GF	Gunning Fog
ARI	Automated Readability Index
CLI	Coleman–Liau Index
USDHHS	US Department of Health and Human Services
SD	Standard Deviation
SE	Standard Errors
ANOVA	Analysis of Variance
