# Supplementary material for: Evaluation of Large Language Models in Generating Physical Exercise Rehabilitation Programs for Musculoskeletal Disorders Across Multiple Clinical Scenarios
Source: Healthcare (Basel). 2026 Aug 4;14(15):2389. doi: 10.3390/healthcare14152389 (PMC13465567; doi:10.3390/healthcare14152389)
Supplement: Supplementary file 1 [file healthcare-14-02389-s001.zip › Supporting Information S1.pdf]

# Supporting information S1

**Figure S1** Search strategy for acquiring questions prior to exclusion in Scenario-1 stage
**Table S1** Questions provided to LLMs in Scenario-1 stage
**Table S2** Excluded questions in Scenario-1 stage
**Table S3** Basic questions provided to LLMs in Scenario-2 stage
**Figure S2** Format of patient information of Scenario-3 questions
**Table S4** Summary of patients' information in Scenario-3 stage
**Table S5** Example prompt types
**Figure S3** Anonymization strategy in Scenario-3 stage
**Table S6** LLMs' generated results categories with their evaluation strategy
**Table S7** Questions used to conduct human and LLMs evaluation
**Table S8** Tukey's Test Results of Readability Scores
**Table S9** Tukey's Test Results of DISCERN Scores
**Table S10** Linear mixed-effects model analysis of LLM-generated response scores
**Table S11** Tukey's Test Results of Readability Scores

Figure S1 Search strategy for acquiring questions prior to exclusion in Scenario-1

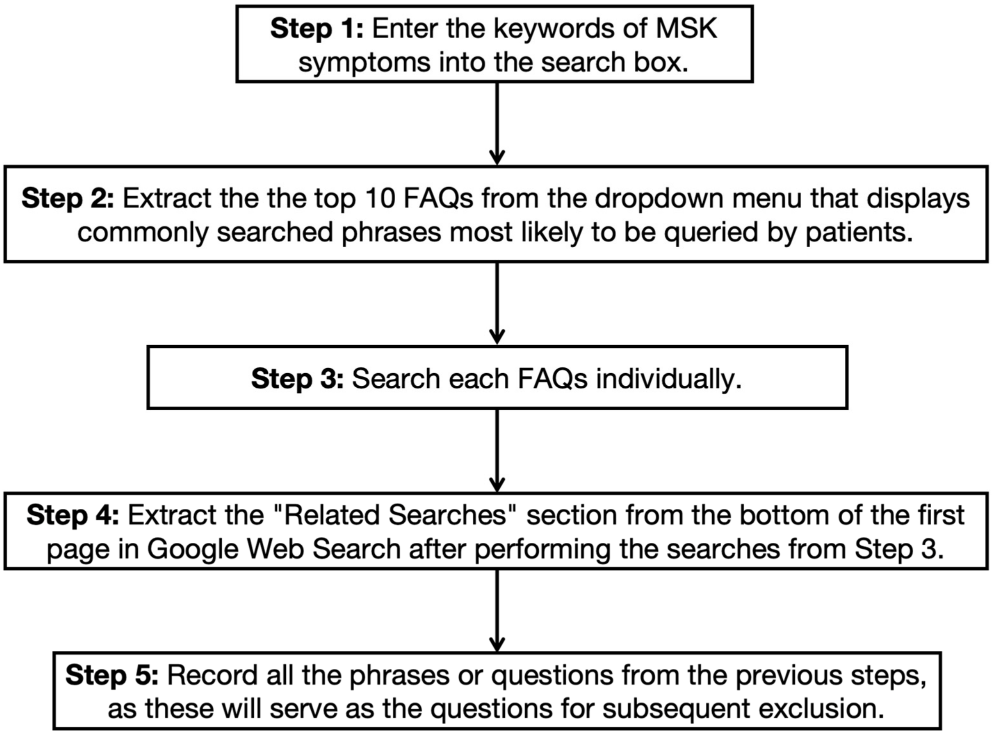


Table S1 Selected questions without prompt provided to LLMs in Scenario-1 stage

| Disease | Selected FAQs of each disease |
|---|---|
| **Low back pain** | physiotherapy exercises for lower back pain<br>how to relieve lower back pain fast<br>what are the 3 simple exercises for back pain<br>13 stretches for lower back pain<br>morning stretches for lower back pain<br>core strengthening exercises for lower back pain<br>how to relieve lower back pain while sleeping<br>how to relieve back pain fast at home<br>how to relieve severe back pain during pregnancy<br>how to relieve severe lower back pain |
| **Shoulder pain** | **SOLUTIONS TO SHOULDER PAIN FROM SLEEPING ON SIDE**<br>**SHOULDER IMPINGEMENT EXERCISES**<br>**HOW TO GET RID OF SHOULDER PAIN AFTER WAKING UP**<br>**SHOULDER PAIN WEIGHT LIFTING RECOVERY**<br>**SHOULDER PAIN TREATMENT**<br>**ROTATOR CUFF EXERCISES FOR ELDERLY**<br>**QUICK RELIEF FROM SHOULDER PAIN**<br>**PHYSIOTHERAPY EXERCISES FOR SHOULDER PAIN** |

| | |
|---|---|
| | **SHOULDER STRENGTHENING EXERCISES AT HOME**<br>**SHOULDER PAIN RELIEF** |
| **Neck pain** | neck exercises for stiff neck<br>**neck strengthening exercises**<br>**nhs neck exercises for arthritis**<br>**exercises to avoid with neck pain**<br>**neck pain treatment**<br>**neck pain exercises**<br>**how to sleep with neck pain on left side**<br>**how to relieve severe neck pain on left side**<br>**how to relieve neck pain from sleeping**<br>**how to cure neck pain fast** |
| **Knee osteoarthritis** | what is the best treatment for osteoarthritis in knees<br>**knee arthritis exercises to avoid osteoarthritis knee exercises nhs**<br>**exercises for knee osteoarthritis video**<br>**quad strengthening exercises for arthritic knees**<br>**physiotherapy exercises for knee osteoarthritis**<br>**knee osteoarthritis exercises for elderly**<br>**how to prevent knee arthritis from getting worse**<br>**what is the best exercise for arthritic knees**<br>**stretching exercises for osteoarthritis**<br>**chair exercises for arthritic knees** |
| **Hip osteoarthritis** | stretching exercises for arthritis in hips<br>**hip arthritis exercises for elderly**<br>**hip exercises for arthritis from the mayo clinic**<br>**hip pain exercises to avoid**<br>**hip osteoarthritis exercises to avoid**<br>**what is the best exercise for osteoarthritis of the hip**<br>**physical therapy exercises for hip arthritis**<br>**treatment for hip arthritis without surgery**<br>**how can i prevent my hip arthritis from getting worse?**<br>**nhs physio exercises for hip pain** |

Table S2 Excluded questions in Scenario-1 stage

| **Disorders** | **Excluded Questions** |
|---|---|
| **Hip OA** | **STAGES OF OSTEOARTHRITIS OF THE HIP**<br>**WHERE IS HIP ARTHRITIS PAIN FELT**<br>**HIP ARTHRITIS TEST**<br>**LEFT HIP OSTEOARTHRITIS SYMPTOMS**<br>**HOW SERIOUS IS OSTEOARTHRITIS OF THE HIP**<br>**STAGES OF OSTEOARTHRITIS OF THE HIP NHS**<br>**BONE-ON BONE HIP PAIN SYMPTOMS**<br>**EARLY HIP ARTHRITIS SYMPTOMS**<br>**STAGES OF OSTEOARTHRITIS OF THE HIP**<br>**WHERE IS HIP ARTHRITIS PAIN FELT** |

| | |
|---|---|
| | **END-STAGE HIP OSTEOARTHRITIS<br>STAGES OF OSTEOARTHRITIS OF THE HIP NHS<br>BONE-ON BONE HIP PAIN SYMPTOMS<br>HOW SERIOUS IS OSTEOARTHRITIS OF THE HIP<br>HIP OSTEOARTHRITIS EXERCISES PDF<br>IS WALKING GOOD FOR HIP ARTHRITIS<br>STAGES OF OSTEOARTHRITIS OF THE HIP<br>HOW SERIOUS IS OSTEOARTHRITIS OF THE HIP<br>WHERE IS HIP ARTHRITIS PAIN FELT<br>HIP ARTHRITIS PAIN DOWN LEG<br>BONE-ON BONE HIP PAIN SYMPTOMS<br>HIP OSTEOARTHRITIS EXERCISES PDF<br>ICD-10 CODE FOR BILATERAL HIP PAIN<br>RIGHT HIP PAIN ICD-10<br>M16.0 ICD 10<br>M16.11 ICD 10<br>ICD 10 CODE FOR OSTEOARTHRITIS HIP UNSPECIFIED<br>RIGHT TOTAL HIP ARTHROPLASTY ICD-10<br>BILATERAL PRIMARY OSTEOARTHRITIS OF HIP<br>LEFT HIP REPLACEMENT ICD-10<br>STAGES OF OSTEOARTHRITIS OF THE HIP<br>WHAT DOES IT FEEL LIKE TO HAVE ARTHRITIS IN YOUR HIP<br>WHERE IS HIP ARTHRITIS PAIN FELT<br>HIP ARTHRITIS TEST<br>DEGENERATIVE ARTHRITIS HIP SYMPTOMS<br>OSTEOARTHRITIS HIP PAIN<br>REFERRED KNEE PAIN FROM HIP<br>KNEE AND HIP PAIN ON ONE SIDE<br>SUDDEN ONSET HIP AND KNEE PAIN<br>KNEE PAIN CAUSED BY HIP ALIGNMENT<br>WHAT CAUSES HIP PAIN AND KNEE PAIN AT THE SAME TIME<br>KNEE AND HIP PAIN AT NIGHT<br>MEDIAL KNEE PAIN REFERRED FROM HIP<br>SYMPTOMS OF HIP AND KNEE OSTEOARTHRITIS<br>END-STAGE HIP OSTEOARTHRITIS<br>HIP OSTEOARTHRITIS PAIN<br>HIP OSTEOARTHRITIS SYMPTOMS<br>MUSCLES AFFECTED BY HIP OSTEOARTHRITIS** |
| **Knee OA** | **EARLY KNEE ARTHRITIS SYMPTOMS<br>WHAT ARE THE 4 STAGES OF** |

| | |
|---|---|
| | **OSTEOARTHRITIS KNEE**<br>**LIVING WITH OSTEOARTHRITIS OF THE KNEE**<br>**BILATERAL KNEE OSTEOARTHRITIS TREATMENT**<br>**BILATERAL PRIMARY OSTEOARTHRITIS OF KNEE**<br>**M17.12 ICD 10**<br>**ICD-10 RIGHT KNEE PAIN**<br>**M17.11 ICD 10**<br>**M17.9 ICD 10**<br>**BILATERAL KNEE PAIN ICD-10**<br>**M17.0 ICD 10**<br>**ICD-10 CODE FOR RIGHT KNEE REPLACEMENT**<br>**LIVING WITH OSTEOARTHRITIS OF THE KNEE**<br>**OSTEOARTHRITIS KNEE SYMPTOMS**<br>**OSTEOARTHRITIS KNEE EXERCISES PDF**<br>**IS WALKING GOOD FOR ARTHRITIS IN THE KNEE**<br>**KNEE OSTEOARTHRITIS EXERCISES FOR ELDERLY PDF**<br>**OSTEOARTHRITIS KNEE SYMPTOMS**<br>**EXERCISES FOR ARTHRITIC KNEES AND HIPS**<br>**PHYSIOTHERAPY EXERCISES FOR KNEE PAIN PDF**<br>**SEATED KNEE EXERCISES PDF**<br>**KNEE OSTEOARTHRITIS EXERCISES PDF NHS**<br>**NHS KNEE EXERCISES PDF**<br>**ADVANCED KNEE STRENGTHENING EXERCISES PDF**<br>**PHYSIOTHERAPY EXERCISES FOR KNEE PAIN PDF**<br>**KNEE OSTEOARTHRITIS EXERCISES FOR ELDERLY PDF**<br>**ADVANCED KNEE STRENGTHENING EXERCISES PDF**<br>**PHYSIOTHERAPY EXERCISES FOR KNEE PAIN PDF**<br>**SEATED KNEE EXERCISES PDF**<br>**NHS KNEE EXERCISES PDF**<br>**HIP AND KNEE EXERCISES PDF**<br>**KNEE OSTEOARTHRITIS EXERCISES FOR ELDERLY PDF**<br>**OSTEOARTHRITIS X RAY FINDINGS**<br>**OSTEOARTHRITIS MRI FINDINGS**<br>**KNEE OSTEOARTHRITIS RADIOPAEDIA** |

| | |
|---|---|
| | MRI GRADING OF KNEE OSTEOARTHRITIS<br>OSTEOARTHRITIS RADIOLOGY GRADING<br>OSTEOARTHRITIS X RAY FINDINGS<br>KNEE PAIN SPREADING THROUGHOUT LEG<br>CAN KNEE ARTHRITIS CAUSE CALF PAIN<br>CAN KNEE ARTHRITIS CAUSE THIGH PAIN<br>WHAT CAN BE DONE FOR ARTHRITIS IN THE KNEE<br>FRONT OF KNEE CRAMPS<br>OSTEOARTHRITIS KNEE SYMPTOMS<br>SEVERE OSTEOARTHRITIS KNEE<br>ARTHRITIS KNEE PAIN RADIATING DOWN SHIN<br>TYPES OF KNEE BRACES FOR OSTEOARTHRITIS<br>BEST KNEE BRACE FOR OSTEOARTHRITIS<br>ORTHOPEDIC KNEE BRACE<br>KNEE BRACE FOR ARTHRITIS PLUS SIZE<br>UNLOADER KNEE BRACE FOR OSTEOARTHRITIS<br>BEST KNEE SUPPORT FOR ARTHRITIS<br>BEST UNLOADER KNEE BRACE FOR OSTEOARTHRITIS<br>KNEE SUPPORT FOR ARTHRITIS AMAZON |
| **Low Back Pain** | WHAT ORGANS CAN CAUSE LOWER BACK PAIN<br>WHEN SHOULD I BE WORRIED ABOUT LOWER BACK PAIN<br>TYPES OF BACK PAIN<br>SUDDEN SEVERE LOWER BACK PAIN FEMALE<br>SUDDEN SEVERE LOWER BACK PAIN CAN'T MOVE<br>VERTEBROGENIC LOW BACK PAIN<br>CHRONIC BACK PAIN ICD-10<br>LUMBAGO ICD-10<br>UPPER BACK PAIN ICD-10<br>THORACIC BACK PAIN ICD-10<br>MID BACK PAIN ICD-10<br>M54.5 ICD 10<br>DORSALGIA ICD-10<br>WHAT IS THE BEST MEDICINE FOR LOWER BACK PAIN?<br>CAUSES OF BACK PAIN IN FEMALE<br>WHY DOES MY LOWER BACK HURT ALL OF A SUDDEN<br>WHEN SHOULD I BE WORRIED ABOUT LOWER BACK PAIN<br>SUDDEN SEVERE LOWER BACK PAIN |

| | |
|---|---|
| | FEMALE<br>DULL PAIN IN LOWER BACK FEMALE<br>WHEN DOES BACK PAIN START IN PREGNANCY<br>BACK PAIN DURING PREGNANCY 3RD TRIMESTER<br>WHEN SHOULD I WORRY ABOUT BACK PAIN IN PREGNANCY<br>LOWER BACK PAIN IN EARLY PREGNANCY 2 WEEKS<br>PREGNANCY BACK PAIN RELIEF SECOND TRIMESTER<br>HOW TO RELIEVE BACK PAIN DURING PREGNANCY WHILE SLEEPING<br>LOWER BACK PAIN EARLY PREGNANCY 4 WEEKS<br>SHARP BACK PAIN DURING PREGNANCY<br>HOW LONG SHOULD YOU LAY ON A HEATING PAD FOR BACK PAIN<br>BEST HEATING PAD FOR LOWER BACK PAIN<br>WHAT IS BETTER FOR LOWER BACK PAIN HEAT OR COLD?<br>ICE MAKES BACK PAIN WORSE<br>ICE OR HEAT FOR LOWER BACK PAIN SCIATICA<br>HEATING PAD FOR LOWER BACK PAIN WALMART<br>BEST HEATING PAD FOR BACK PAIN<br>HEATING PAD WITH MASSAGE FOR LOWER BACK PAIN<br>LOWER BACK PAIN IN EARLY PREGNANCY 2 WEEKS<br>WHEN SHOULD I WORRY ABOUT BACK PAIN IN PREGNANCY<br>WHEN DOES BACK PAIN START IN PREGNANCY<br>EARLY PREGNANCY BACK PAIN LOCATION<br>HOW TO RELIEVE SEVERE BACK PAIN DURING PREGNANCY<br>UPPER BACK PAIN EARLY PREGNANCY 4 WEEKS<br>LOWER BACK PAIN PREGNANCY THIRD TRIMESTER<br>MIDDLE BACK PAIN EARLY PREGNANCY |
| **Neck Pain** | CAUSES OF BACK & NECK PAIN IN FEMALE<br>NECK PAIN AND NEUROLOGICAL SYMPTOMS<br>BACK OF NECK PAIN COVID |

NECK PAIN WHEN TILTING HEAD BACK
WHAT IS THE BEST PAINKILLER FOR NECK PAIN
WHAT IS THE BEST TREATMENT FOR NECK AND SHOULDER PAIN?
CAUSES OF BACK & NECK PAIN IN FEMALE
TYPES OF NECK PAIN
NECK PAIN CAUSES
WOKE UP WITH NECK PAIN CAN'T TURN HEAD
HOW LONG DOES NECK PAIN LAST FROM SLEEPING WRONG
SHOULDER AND NECK PAIN FROM SLEEPING ON SIDE
NECK PAIN AT NIGHT CANCER
WOKE UP WITH SEVERE NECK PAIN ON LEFT SIDE
WOKE UP WITH NECK PAIN ON RIGHT SIDE
BEST SLEEPING POSITION FOR NECK AND SHOULDER PAIN
NECK STRAIN ICD-10
NECK STIFFNESS ICD-10
THORACIC PAIN ICD-10
LOW BACK PAIN ICD-10
SHOULDER PAIN ICD-10
ICD-10 CERVICALGIA WITH RADICULOPATHY
UPPER BACK PAIN ICD-10
BACK PAIN ICD-10
CERVICOGENIC HEADACHE
CERVICOGENIC HEADACHE TUMOR
HEADACHE IN BACK OF HEAD AND NECK
HOW LONG CAN A CERVICOGENIC HEADACHE LAST
CERVICOGENIC HEADACHE SYMPTOMS
NECK HEADACHE
STIFF NECK AND HEADACHE
RIGHT SIDE SHOULDER AND NECK PAIN ASSOCIATED WITH HEADACHES
WOMAN NECK AND SHOULDER PAIN ON LEFT SIDE
PAIN IN LEFT SIDE OF NECK AND SHOULDER
WEIRD FEELING IN NECK LEFT SIDE
PAIN ON LEFT SIDE OF NECK WHERE PULSE IS
NECK AND SHOULDER PAIN ON LEFT SIDE GOING DOWN ARM
PAIN ON RIGHT SIDE OF NECK

| | |
|---|---|
| | **NECK AND SHOULDER PAIN ON LEFT SIDE AFTER SLEEPING**<br>**BURNING SENSATION ON LEFT SIDE OF NECK AND SHOULDER**<br>**WOMAN NECK AND SHOULDER PAIN ON RIGHT SIDE**<br>**NECK VEIN PAIN RIGHT SIDE**<br>**PAIN IN RIGHT SIDE OF NECK UNDER EAR AND JAW**<br>**SHOULDER AND NECK PAIN ON RIGHT SIDE**<br>**WEIRD FEELING IN NECK RIGHT SIDE**<br>**SHARP PAIN IN RIGHT SIDE OF NECK**<br>**SHOULDER AND NECK PAIN ON RIGHT SIDE HOME REMEDIES**<br>**PAIN IN FRONT OF NECK RIGHT SIDE**<br>**WOKE UP WITH NECK PAIN CAN'T TURN HEAD**<br>**LEFT SIDE OF NECK HURTS AFTER SLEEPING**<br>**SHOULDER AND NECK PAIN FROM SLEEPING ON SIDE**<br>**NECK PAIN AFTER SLEEPING EVERYDAY**<br>**SEVERE NECK PAIN AFTER SLEEPING**<br>**NECK PAIN AT NIGHT CANCER**<br>**HOW LONG DOES NECK PAIN LAST FROM SLEEPING WRONG**<br>**WHY DOES MY NECK HURT WHEN I LAY DOWN ON A PILLOW**<br>**NECK AND SHOULDER PAIN EXERCISES AND STRETCHES**<br>**BEST EXERCISE FOR NECK AND SHOULDER PAIN**<br>**PHYSIOTHERAPY EXERCISES FOR NECK AND SHOULDER PAIN PDF**<br>**CAUSES OF BACK & NECK PAIN IN FEMALE**<br>**CAN NECK PAIN BE A SIGN OF SOMETHING SERIOUS**<br>**NECK PAIN AND NEUROLOGICAL SYMPTOMS**<br>**WHEN A STIFF NECK IS SERIOUS**<br>**WHAT IS THE BEST TREATMENT FOR NECK AND SHOULDER PAIN?** |
| **Shoulder Pain** | **SHOULDER HURTS WHEN SLEEPING ON SIDE REDDIT**<br>**LEFT SHOULDER PAIN AFTER SLEEPING**<br>**NECK AND SHOULDER PAIN FROM SLEEPING ON SIDE**<br>**SHOULDER PAIN AT NIGHT CANCER**<br>**SHOULDER PAIN AT NIGHT RELIEF** |

| | |
|---|---|
| | **BEST PAINKILLER FOR SEVERE SHOULDER PAIN**<br>**LEFT SHOULDER PAIN IN WOMEN**<br>**RIGHT SHOULDER PAIN WITHOUT INJURY**<br>**SHOULDER PAIN WHEN LIFTING ARM**<br>**SHOULDER PAIN REASONS**<br>**SUDDEN SHOULDER PAIN WITHOUT INJURY**<br>**RIGHT SHOULDER PAIN IN WOMEN**<br>**CAUSES OF SHOULDER PAIN IN FEMALE**<br>**WHAT CAUSES PAIN IN BOTH SHOULDERS AND UPPER ARMS**<br>**RIGHT SHOULDER PAIN IN WOMEN**<br>**LEFT SHOULDER PAIN IN WOMEN**<br>**LEFT SHOULDER PAIN WITHOUT INJURY**<br>**RIGHT SHOULDER PAIN WITHOUT INJURY**<br>**PHYSIOTHERAPY EXERCISES FOR SHOULDER AND ARM PAIN**<br>**NECK AND SHOULDER PAIN EXERCISES AND STRETCHES**<br>**LEFT SHOULDER PAIN BENCH PRESS REDDIT**<br>**COMMON BENCH PRESS INJURIES SHOULDER**<br>**BENCH PRESS SHOULDER IMPINGEMENT**<br>**ROTATOR CUFF BENCH PRESS**<br>**AC JOINT PAIN BENCH PRESS**<br>**SHOULDER PAIN WHEN BENCHING BUT NOT PRESSING**<br>**BENCH PRESS INJURY CHEST**<br>**SHOULDER PAIN WHEN PRESSING**<br>**MY UPPER ARM HURTS WHEN I RAISE IT**<br>**WHY DOES MY SHOULDER HURT WHEN I LIFT MY ARM TO THE SIDE**<br>**SHOULDER PAIN WHEN CROSSING ARM OVER CHEST**<br>**WOKE UP WITH SHOULDER PAIN CAN'T LIFT ARM**<br>**CAN'T PUT ARM BEHIND BACK WITHOUT PAIN**<br>**CAN'T LIFT ARM ABOVE SHOULDER WITHOUT PAIN**<br>**LEFT SHOULDER PAIN WITHOUT INJURY**<br>**LEFT SHOULDER PAIN CAUSES**<br>**SHOULDER PAIN WHEN LIFTING ARM**<br>**WOMAN PAIN IN LEFT SHOULDER AND NECK**<br>**RIGHT SHOULDER PAIN IN WOMEN**<br>**CAN I STILL WORKOUT WITH SHOULDER PAIN** |

| | |
|---|---|
| | **SHOULDER PAIN 2 DAYS AFTER WORKOUT**<br>**SHOULDER PAIN AFTER LIFTING SOMETHING HEAVY**<br>**LEFT SHOULDER PAIN GYM REDDIT**<br>**SHOULDER PAIN AFTER CHEST WORKOUT**<br>**SHOULDER BLADE PAIN AFTER WORKOUT**<br>**WEIGHT LIFTING FOR SHOULDER PAIN**<br>**SHOULDER PAIN WHEN LIFTING ARM**<br>**SHOULDER PAIN REASONS**<br>**BEST PAINKILLER FOR SEVERE SHOULDER PAIN**<br>**LEFT SHOULDER PAIN WITHOUT INJURY**<br>**LEFT SHOULDER PAIN IN WOMEN**<br>**SHOULDER PAIN FROM SLEEPING** |

Table S3 Basic questions provided to LLMs in Scenario-2 stage

| | |
|---|---|
| **Low back pain** | **I WAS DIAGNOSED AS RADICULAR LOW BACK PAIN, PLEASE ACCORDING TO THE FITT-VP PRINCIPLE GIVE ME A PHYSICAL EXERCISE REHABILITATION PLAN, AND LIST THE GOAL AND REASON OF EACH STAGE.** |
| | **I WAS DIAGNOSED AS NON-RADICULAR ACUTE LOW BACK PAIN, PLEASE ACCORDING TO THE FITT-VP PRINCIPLE GIVE ME A PHYSICAL EXERCISE REHABILITATION PLAN, AND LIST THE GOAL AND REASON OF EACH STAGE.**<br>**I WAS DIAGNOSED AS NON-RADICULAR CHRONIC LOW BACK PAIN, PLEASE ACCORDING TO THE FITT-VP PRINCIPLE GIVE ME A PHYSICAL EXERCISE REHABILITATION PLAN, AND LIST THE GOAL AND REASON OF EACH STAGE.** |
| **Shoulder pain** | **I WAS DIAGNOSED AS ROTATOR CUFF DISORDER, PLEASE ACCORDING TO THE FITT-VP PRINCIPLE GIVE ME A PHYSICAL EXERCISE REHABILITATION PLAN, AND LIST THE GOAL AND REASON OF EACH STAGE.** |
| | **I WAS DIAGNOSED AS FROZEN SHOULDER, PLEASE ACCORDING TO THE FITT-VP PRINCIPLE GIVE ME A PHYSICAL EXERCISE REHABILITATION PLAN, AND LIST THE GOAL AND REASON OF EACH STAGE.**<br>**I WAS DIAGNOSED AS PERIARTHRITIS OF THE SHOULDER, PLEASE ACCORDING TO THE FITT-VP PRINCIPLE GIVE ME A PHYSICAL EXERCISE REHABILITATION PLAN, AND LIST THE GOAL AND REASON OF EACH STAGE.**<br>**I WAS DIAGNOSED AS THE TRAUMATIC DISLOCATION OF THE SHOULDER, PLEASE** |

| | |
|---|---|
| | ACCORDING TO THE FITT-VP PRINCIPLE GIVE ME A PHYSICAL EXERCISE REHABILITATION PLAN, AND LIST THE GOAL AND REASON OF EACH STAGE.<br>I WAS DIAGNOSED AS THE ACROMIOCLAVICULAR DISEASE, PLEASE ACCORDING TO THE FITT-VP PRINCIPLE GIVE ME A PHYSICAL EXERCISE REHABILITATION PLAN, AND LIST THE GOAL AND REASON OF EACH STAGE. |
| **Neck pain** | I WAS DIAGNOSED AS THE FACET JOINT OSTEOARTHRITIS(FJ OA), PLEASE ACCORDING TO THE FITT-VP PRINCIPLE GIVE ME A PHYSICAL EXERCISE REHABILITATION PLAN, AND LIST THE GOAL AND REASON OF EACH STAGE. |
| | I WAS DIAGNOSED AS THE DEGENERATIVE CERVICAL DISC DISEASE, PLEASE ACCORDING TO THE FITT-VP PRINCIPLE GIVE ME A PHYSICAL EXERCISE REHABILITATION PLAN, AND LIST THE GOAL AND REASON OF EACH STAGE.<br>I WAS DIAGNOSED AS DEGENERATIVE CERVICAL SPONDYLOSIS, PLEASE ACCORDING TO THE FITT-VP PRINCIPLE GIVE ME A PHYSICAL EXERCISE REHABILITATION PLAN, AND LIST THE GOAL AND REASON OF EACH STAGE.<br>I WAS DIAGNOSED AS DEGENERATIVE CERVICAL SPONDYLOSIS, PLEASE ACCORDING TO THE FITT-VP PRINCIPLE GIVE ME A PHYSICAL EXERCISE REHABILITATION PLAN, AND LIST THE GOAL AND REASON OF EACH STAGE.<br>I WAS DIAGNOSED AS WHIPLASH ASSOCIATED DISORDERS (WAD) CATEGORY I, PLEASE ACCORDING TO THE FITT-VP PRINCIPLE GIVE ME A PHYSICAL EXERCISE REHABILITATION PLAN, AND LIST THE GOAL AND REASON OF EACH STAGE.<br>I WAS DIAGNOSED AS WHIPLASH ASSOCIATED DISORDERS (WAD) CATEGORY II, PLEASE ACCORDING TO THE FITT-VP PRINCIPLE GIVE ME A PHYSICAL EXERCISE REHABILITATION PLAN, AND LIST THE GOAL AND REASON OF EACH STAGE.<br>I WAS DIAGNOSED AS NONSPECIFIC NECK PAIN, PLEASE ACCORDING TO THE FITT-VP PRINCIPLE GIVE ME A PHYSICAL EXERCISE REHABILITATION PLAN, AND LIST THE GOAL AND REASON OF EACH STAGE. |
| **Knee osteoarthritis** | I WAS DIAGNOSED AS KELLGREN-LAWRENCE GRADE 1 KNEE OSTEOARTHRITIS, PLEASE ACCORDING TO THE FITT-VP PRINCIPLE GIVE ME A PHYSICAL EXERCISE REHABILITATION PLAN, |

| | |
|---|---|
| | **AND LIST THE GOAL AND REASON OF EACH STAGE.** |
| | **I WAS DIAGNOSED AS KELLGREN-LAWRENCE GRADE 2 KNEE OSTEOARTHRITIS, PLEASE ACCORDING TO THE FITT-VP PRINCIPLE GIVE ME A PHYSICAL EXERCISE REHABILITATION PLAN, AND LIST THE GOAL AND REASON OF EACH STAGE.**<br>**I WAS DIAGNOSED AS KELLGREN-LAWRENCE GRADE 3 KNEE OSTEOARTHRITIS, PLEASE ACCORDING TO THE FITT-VP PRINCIPLE GIVE ME A PHYSICAL EXERCISE REHABILITATION PLAN, AND LIST THE GOAL AND REASON OF EACH STAGE.**<br>**I WAS DIAGNOSED AS KELLGREN-LAWRENCE GRADE 4 KNEE OSTEOARTHRITIS, PLEASE ACCORDING TO THE FITT-VP PRINCIPLE GIVE ME A PHYSICAL EXERCISE REHABILITATION PLAN, AND LIST THE GOAL AND REASON OF EACH STAGE.** |
| **Hip osteoarthritis** | **I WAS DIAGNOSED AS KELLGREN-LAWRENCE GRADE 1 HIP OSTEOARTHRITIS, PLEASE ACCORDING TO THE FITT-VP PRINCIPLE GIVE ME A PHYSICAL EXERCISE REHABILITATION PLAN, AND LIST THE GOAL AND REASON OF EACH STAGE.** |
| | **I WAS DIAGNOSED AS KELLGREN-LAWRENCE GRADE 2 HIP OSTEOARTHRITIS, PLEASE ACCORDING TO THE FITT-VP PRINCIPLE GIVE ME A PHYSICAL EXERCISE REHABILITATION PLAN, AND LIST THE GOAL AND REASON OF EACH STAGE.**<br>**I WAS DIAGNOSED AS KELLGREN-LAWRENCE GRADE 3 HIP OSTEOARTHRITIS, PLEASE ACCORDING TO THE FITT-VP PRINCIPLE GIVE ME A PHYSICAL EXERCISE REHABILITATION PLAN, AND LIST THE GOAL AND REASON OF EACH STAGE.**<br>**I WAS DIAGNOSED AS KELLGREN-LAWRENCE GRADE 4 HIP OSTEOARTHRITIS, PLEASE ACCORDING TO THE FITT-VP PRINCIPLE GIVE ME A PHYSICAL EXERCISE REHABILITATION PLAN, AND LIST THE GOAL AND REASON OF EACH STAGE.** |

Figure S2 Format of Patient Information of Scenario-3 Questions

# Patient Information Form

- Personal Details
  Full Name: ________________________________________
  Date of Birth (mm/dd/yyyy): __________________________
  Sex: __________________(Male / Female / Other Female)
  Height (in cm): _____________________________________
  Weight (in kg): _____________________________________

- Medical History
  Chief medical problem and diagnosis.
  Pain Levels (please rate on a scale of 0-10, with 0 being no pain and 10 being the worst possible pain): ______________
  Functional Limitations (please describe any specific activities that are limited due to Low back pain): _________________
  Comorbidities (please list any other medical conditions): _________________
  Contraindications (please list any specific exercises or activities that should be avoided due to other medical conditions or concerns): _________________

- Goals and Preferences
  Rehabilitation Goals (please list the primary goals you would like to achieve with the rehabilitation program, e.g., pain reduction, improved mobility, increased strength): _________________________
  Exercise Preferences (please list any specific exercises or activities you enjoy or would like to include in the program): _________________________

- Equipment and Resources
  Available Equipment (please list any exercise equipment you have access to, e.g., resistance bands, weights, treadmill, stationary bike): _________________________
  Exercise Environment (please describe the location where you will perform the exercises, e.g., home, gym, outdoors, physical therapy clinic): _________________

- Work: _________________

- Living environment: _________________

Table S4 Summary of patients' information in Scenario-3 stage

| Patient ID | Age | Gender | Height | Weight | Pain Level | Functional Limitation | Comorbidities | Contraindications | Exercise Preference | Exercise Equipment | Exercise Environment |
|---|---|---|---|---|---|---|---|---|---|---|---|
| **01** | 24 | Female | 165 | 48 | 2 | NA | NA | NA | Dancing and Yoga | weights, resistance band | Home and Dance studio |
| **02** | 29 | Female | 169 | 56 | 4 | Squatting | NA | NA | Running | Stationary bike and other equipment at gym | Gym and Home |
| **03** | 52 | Male | 170 | 79 | 6 | NA | Previous fracture of right femur treated with internal fixation | NA | Basketball | Weights | Home and Basketball field |
| **04** | 74 | Male | 172 | 74 | 5 | NA | Hypertension, Previous arthroscopic surgery for right hip labrum tear | NA | Volleyball | Resistance band | Gym and Home |
| **05** | 55 | Male | 169 | 74 | 8 | Hip flexion | systemic lupus erythematosus | NA | NA | Resistance band, Weights | Home |
| **06** | 60 | Female | 160 | 63 | 4 | Squatting | Diabetes | NA | Walking | resistance band | Home and park |
| **07** | 65 | Male | 165 | 70 | 3 | Climbing stairs | Hypertension, Atrial fibrillation | NA | Hiking | Resistance band, stationary bike, and | Gym and Home |

| | | | | | | | | | | | |
|---|---|---|---|---|---|---|---|---|---|---|---|
| | | | | | | | | | | other equipment available at Gym | |
| **08** | 91 | Male | 158 | 73 | 4 | Muscle weakness in bilateral lower limb | Coronary artery disease, Lumbar disc herniation | NA | NA | NA | Home |
| **09** | 42 | Female | 175 | 69 | 4 | NA | NA | NA | Swimming and gym-based exercise | Resistance band, weights | Gym and Home |
| **10** | 73 | Male | 178 | 80 | 5 | Limited knee flexion | NA | Previous THA surgery for bilateral hip OA | Soccer | Weights | Home and Soccer field |
| **11** | 32 | Female | 175 | 68 | 3 | NA | NA | NA | Swimming | resistance band, weights | Gym and Home |
| **12** | 20 | Female | 165 | 50 | 6 | Bending down exacerbates pain | Type I diabetes | NA | Dancing | Resistance band | Gym and Home |
| **13** | 85 | Female | 162 | 65 | 4 | Muscle weakness in bilateral lower limb | Hypertension, Coronary artery disease | Unable to swim or running | NA | NA | Home |
| **14** | 59 | Male | 160 | 75 | 5 | NA | Type II diabetes | Unable to Run | Swimming | Resistance band and stationary bike | Gym and Home |
| **15** | 64 | Male | 180 | 80 | 7 | Cannot bend down | Knee OA | NA | Soccer | Weights | Gym and Home |
| **16** | 31 | Female | 170 | 68 | 4 | NA | NA | NA | Gym-based exercise | weights, resistance band | Gym and Home |
| **17** | 54 | Male | 165 | 69 | 2 | NA | Hypertension | NA | NA | NA | Home |
| **18** | 24 | Female | 166 | 43 | 6 | Limited neck flexion | NA | NA | Swimming | Resistance band | Home and swimming pool |
| **19** | 84 | Male | 156 | 60 | 5 | NA | Diabetes, coronary artery disease | NA | NA | NA | NA |
| **20** | 34 | Male | 173 | 69 | 4 | NA | NA | NA | Gym-based exercise | Gym-based equipment | Gym and Home |
| **21** | 51 | Female | 160 | 63 | 7 | Cannot subjectively abduct right shoulder. Passive shoulder movement is intact | NA | NA | NA | Resistance band | Home |
| **22** | 43 | Male | 165 | 69 | 4 | Limited range of motion in left shoulder | NA | NA | Tennis | weights, resistance band | Home, Gym and Tennis field |
| **23** | 61 | Female | 157 | 62 | 6 | Limited abduction and extension | Diabetes | NA | NA | NA | Home |
| **24** | 79 | Male | 162 | 70 | 3 | NA | Hypertension, Lung cancer | NA | NA | Resistance band | Home |
| **25** | 25 | Female | 172 | 65 | 8 | Highly limited range of motion | NA | NA | Badminton | Gym-based equipment | Gym and Home |
| **26** | 22 | Male | 183 | 69 | 3 | NA | NA | NA | Volleyball | Gym-based equipment | Gym and Home |
| **27** | 38 | Female | 165 | 60 | 3 | External rotation of right hip is limited | NA | NA | NA | Resistance band | Home |
| **28** | 64 | Female | 152 | 68 | 8 | Hip flexion | Osteoporosis | Hypertension | NA | Resistance band | Home and park |
| **29** | 19 | Female | 169 | 45 | 7 | Hip abduction and hip external rotation are limited | NA | NA | Dancing | Resistance band | Home |
| **30** | 47 | Female | 155 | 45 | 7 | NA | NA | NA | NA | NA | Home |
| **31** | 35 | Male | 170 | 70 | 4 | Limited knee flexion | NA | NA | Tennis | Gym-based equipment | Gym and Home |
| **32** | 42 | Female | 175 | 85 | 5 | NA | Gout | NA | Hiking | weights, resistance band and runner | Gym and Home |
| **33** | 66 | Female | 150 | 50 | 7 | NA | Diabetes and osteoporosis | NA | Walking | weights, resistance band | Gym and Home |
| **34** | 23 | Female | 165 | 60 | 5 | Cannot squat | NA | NA | Hiking | weights, resistance band | Gym and Home |
| **35** | 52 | Male | 170 | 80 | 7 | NA | NA | NA | Soccer | Weights | Home and Soccer field |
| **36** | 60 | Female | 160 | 60 | 7 | NA | Osteoporosis | Thoracic spine compression fracture | NA | NA | Home |
| **37** | 24 | Male | 160 | 70 | 7 | Cannot bend down and get up from bed without pain | NA | NA | NA | NA | Home |
| **38** | 42 | Female | 170 | 40 | 6 | NA | NA | NA | Aerobic | NA | Gym and Home |
| **39** | 23 | Female | 175 | 80 | 4 | NA | NA | NA | Yoga | weights, resistance band | Gym and Home |
| **40** | 42 | Male | 168 | 85 | 8 | NA | NA | NA | Home-based exercise | weights, resistance band | Home |
| **41** | 24 | Male | 170 | 65 | 5 | NA | NA | NA | NA | Gym-based equipment | Gym and Home |
| **42** | 54 | Female | 164 | 60 | 6 | ROM Limited | NA | NA | Yoga | Gym-based equipment | Gym |
| **43** | 23 | Male | 175 | 74 | 7 | NA | NA | NA | NA | Resistance band | Home |
| **44** | 76 | Male | 170 | 80 | 6 | NA | Hypertension | NA | NA | NA | Home |
| **45** | 39 | Male | 168 | 50 | 4 | NA | NA | NA | Hiking | Gym-based equipment | Gym and Home |
| **46** | 24 | Female | 165 | 65 | 6 | NA | NA | NA | Gym-based exercise | Gym-based equipment | Gym and Home |
| **47** | 24 | Male | 170 | 60 | 3 | NA | NA | NA | Basketball | Gym-based equipment | Gym and Home |
| **48** | 48 | Female | 162 | 70 | 8 | Highly limited range of motion | NA | NA | Yoga | Gym-based equipment | Gym and Home |
| **49** | 41 | Female | 170 | 50 | 6 | Limited abduction | NA | NA | Running | Resistance band, Weights | Home |
| **50** | 45 | Female | 175 | 80 | 4 | NA | NA | NA | Soccer | NA | Home |

Table S5 Example prompt types

| **Prompt Type** | **Example** |
|---|---|
| **None prompt word** | I was diagnosed as Kellgren-Lawrence grade 1 hip |

| | |
|---|---|
| | osteoarthritis, please according to the FITT-VP principle give me a physical exercise rehabilitation plan, and list the goal and reason of each stage. |
| **Identity prompt words** | I am a patient, I was diagnosed as Kellgren-Lawrence grade 1 hip osteoarthritis, please according to the FITT-VP principle give me a physical exercise rehabilitation plan, and list the goal and reason of each stage. |
| **CoT prompt words** | I was diagnosed as Kellgren-Lawrence grade 1 hip osteoarthritis, please according to the FITT-VP principle give me a physical exercise rehabilitation plan, and list the goal and reason of each stage.<br>Perform the task step by step. |

Figure S3 Anonymization strategy in Scenario-3 stage

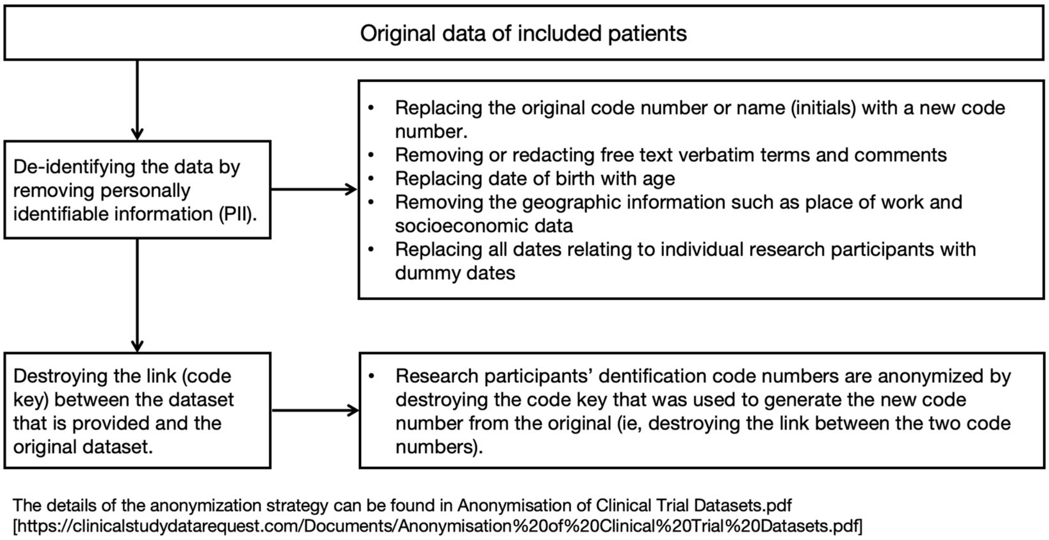


**Table S6** Categories of LLMs' generated results and evaluation strategy

| **LLMs' generated results** | | | **Evaluation strategy** |
|---|---|---|---|
| **Model** | Prompt | Scenario | |
| **ChatGPT-4** | Without Additional Prompt | 1 | Human Evaluation |
| | | 2 | & |
| | | 3 | LLM Evaluation |
| **ChatGPT-4** | With Identity Prompt | 1 | LLM Evaluation |
| | | 2 | & |
| | | 3 | Human Check |
| **ChatGPT-4** | With CoT Prompt | 1 | LLM Evaluation |
| | | 2 | & |
| | | 3 | Human Check |
| **ChatGPT-3.5** | Without Additional Prompt | 1 | LLM Evaluation |
| | | 2 | & |

| | | | |
|---|---|---|---|
| | | 3 | Human Check |
| **ChatGPT-3.5** | With Identity Prompt | 1 | LLM Evaluation & Human Check |
| | | 2 | |
| | | 3 | |
| **ChatGPT-3.5** | With CoT Prompt | 1 | LLM Evaluation & Human Check |
| | | 2 | |
| | | 3 | |
| **ChatGPT-4o** | Without Additional Prompt | 1 | LLM Evaluation & Human Check |
| | | 2 | |
| | | 3 | |
| **ChatGPT-4o** | With Identity Prompt | 1 | LLM Evaluation & Human Check |
| | | 2 | |
| | | 3 | |
| **ChatGPT-4o** | With CoT Prompt | 1 | LLM Evaluation & Human Check |
| | | 2 | |
| | | 3 | |
| **Claude** | Without Additional Prompt | 1 | LLM Evaluation & Human Check |
| | | 2 | |
| | | 3 | |
| **Claude** | With Identity Prompt | 1 | LLM Evaluation & Human Check |
| | | 2 | |
| | | 3 | |
| **Claude** | With CoT Prompt | 1 | LLM Evaluation & Human Check |
| | | 2 | |
| | | 3 | |

Table S7 Questions used to conduct human and LLMs evaluation

| Question ID | Questions |
|---|---|
| **Q1** | Q1. Are the aims of the publication clear?<br><br>Please evaluate this aspect of the publication's reliability using a 1-5 scoring system, where:<br>1 = Strongly diagree<br>2 = Diagree<br>3 = Neutral<br>4 = Agree<br>5 = Strongly agree<br><br>When assessing, consider the following criteria:<br><br>Is there a clear indication at the beginning of the publication about its subject matter? If yes, the score should be high.<br>Does the publication clearly state what topics it intends to cover and which topics are excluded? If yes, the score should be high.<br>Does the publication specify who might find it useful? If yes, the score should be high.<br>Provide your score (1, 2, 3, 4 or 5) and explain your reasoning. Include specific examples from the publication that support your score, or note any missing content that influenced your decision. |

| | |
|---|---|
| | Output the final answer in json:<br>Score:[numberic],Explanation:[Words] |
| **Q2** | Q2. Does the publication’ s achieve its aims?<br><br>Please evaluate this aspect of the publication's reliability using a 1-5 scoring system, where:<br>1 = Strongly diagree<br>2 = Diagree<br>3 = Neutral<br>4 = Agree<br>5 = Strongly agree<br><br>When assessing, consider the following criteria:<br><br>Does the publication provides the information it aimed to as indicated clearly at the beginning of the publication about its subject matter? If yes, the score should be high.<br>Provide your score (1, 2, 3, 4 or 5) and explain your reasoning. Include specific examples from the publication that support your score, or note any missing content that influenced your decision.<br>Output the final answer in json:<br>Score:[numberic],Explanation:[Words] |
| **Q3** | Q3. Is the publication relevant?<br><br>Please evaluate this aspect of the publication's reliability using a 1-5 scoring system, where:<br>1 = Strongly diagree<br>2 = Diagree<br>3 = Neutral<br>4 = Agree<br>5 = Strongly agree<br><br>When assessing, consider the following criteria:<br><br>Does the publication addresses the questions that readers might ask? If yes, the score should be high.<br>Does the recommendations and suggestions concerning treatment choices in the publication are realistic or appropriate? If yes, the score should be high.<br>Provide your score (1, 2, 3, 4 or 5) and explain your reasoning. Include specific examples from the publication that support your score, or note any missing content that influenced your decision.<br>Output the final answer in json:<br>Score:[numberic],Explanation:[Words] |
| **Q4** | Q4. Is it clear what sources of information were used to compile the publication (other than the author or producer)?<br><br>Please evaluate this aspect of the publication's reliability using a 1-5 scoring system, where: |

| | |
|---|---|
| | 1 = Strongly diagree<br>2 = Diagree<br>3 = Neutral<br>4 = Agree<br>5 = Strongly agree<br><br>When assessing, consider the following criteria:<br><br>Are the main claims or statements made about treatment choices in the publication accompanied by references to the sources used as evidence, such as research studies or expert opinions? If yes, the score should be high.<br>Does the publication include sources such as a bibliography or reference list, or the addresses of the experts or organizations quoted? If yes, the score should be high.<br>To receive a top score of '5,' the publication must include both citations for the sources used as evidence and a bibliography or reference list, as well as the contact information for the cited experts or organizations.<br>Provide your score (1, 2, 3, 4 or 5) and explain your reasoning. Include specific examples from the publication that support your score, or note any missing content that influenced your decision.<br>Output the final answer in json:<br>Score:[numberic],Explanation:[Words] |
| **Q5** | Q5. Is it clear when the information used or reported in the publication was produced?<br><br>Please evaluate this aspect of the publication's reliability using a 1-5 scoring system, where:<br>1 = Strongly diagree<br>2 = Diagree<br>3 = Neutral<br>4 = Agree<br>5 = Strongly agree<br><br>When assessing, consider the following criteria:<br><br>Does the publication include the dates of the main sources of information, the date of any revisions (excluding reprint dates), and the copyright date of the publication? If yes, the score should be high.<br>To achieve a perfect score of '5,' the publication must include the dates associated with its main sources of information.<br>Provide your score (1, 2, 3, 4 or 5) and explain your reasoning. Include specific examples from the publication that support your score, or note any missing content that influenced your decision.<br>Output the final answer in json:<br>Score:[numberic],Explanation:[Words] |
| **Q6** | Q6. Is the publication balanced and unbiased? |

| | |
|---|---|
| | Please evaluate this aspect of the publication's reliability using a 1-5 scoring system, where:<br>1 = Strongly diagree<br>2 = Diagree<br>3 = Neutral<br>4 = Agree<br>5 = Strongly agree<br><br>When assessing, consider the following criteria:<br><br>Does the publication demonstrate that a variety of information sources were used to compile it? If yes, the score should be high.<br>Does the publication focus solely on the advantages or disadvantages of one specific treatment without referencing other available options? If yes, the score should be low.<br>Does the publication mainly uses evidence from single cases, which may not be representative of typical responses to a treatment or of people with this condition, and presents information in a sensational, emotive, or alarmist manner? If yes, the score should be low.<br>Provide your score (1, 2, 3, 4 or 5) and explain your reasoning. Include specific examples from the publication that support your score, or note any missing content that influenced your decision.<br>Output the final answer in json:<br>Score:[numberic],Explanation:[Words] |
| **Q7** | Q7. Does the publication provide  details of additional sources of support and information?<br><br>Please evaluate this aspect of the publication's reliability using a 1-5 scoring system, where:<br>1 = Strongly diagree<br>2 = Diagree<br>3 = Neutral<br>4 = Agree<br>5 = Strongly agree<br><br>When assessing, consider the following criteria:<br><br>Does the publication recommend further reading or provide details of other organizations that offer advice and information about the condition and treatment options? If yes, the score should be high.<br>Provide your score (1, 2, 3, 4 or 5) and explain your reasoning. Include specific examples from the publication that support your score, or note any missing content that influenced your decision.<br>Output the final answer in json:<br>Score:[numberic],Explanation:[Words] |
| **Q8** | Q8. Does the publication refer to areas of uncertainty?<br><br>Please evaluate this aspect of the publication's reliability using a 1-5 scoring system, where: |

| | |
|---|---|
| | 1 = Strongly diagree<br>2 = Diagree<br>3 = Neutral<br>4 = Agree<br>5 = Strongly agree<br><br>When assessing, consider the following criteria:<br><br>Does the publication discuss gaps in knowledge or differences in expert opinion concerning treatment choices? If yes, the score should be high.<br>Does the publication imply that a treatment choice affects everyone in the same way? If yes, the score should be low.<br>Provide your score (1, 2, 3, 4 or 5) and explain your reasoning. Include specific examples from the publication that support your score, or note any missing content that influenced your decision. Output the final answer in json:<br>Score:[numberic],Explanation:[Words] |
| **Q9** | Q9. Does it describe how each treatment works?<br><br>Please evaluate this aspect of the publication's reliability using a 1-5 scoring system, where:<br>1 = Strongly diagree<br>2 = Diagree<br>3 = Neutral<br>4 = Agree<br>5 = Strongly agree<br><br>When assessing, consider the following criteria:<br><br>Does the publication describe how a treatment acts on the body to achieve its effects? If yes, the score should be high.<br>Provide your score (1, 2, 3, 4 or 5) and explain your reasoning. Include specific examples from the publication that support your score, or note any missing content that influenced your decision. Output the final answer in json:<br>Score:[numberic],Explanation:[Words] |
| **Q10** | Q10. Does the publication describe the benefits of each treatment?<br><br>Please evaluate this aspect of the publication's reliability using a 1-5 scoring system, where:<br>1 = Strongly diagree<br>2 = Diagree<br>3 = Neutral<br>4 = Agree<br>5 = Strongly agree<br><br>When assessing, consider the following criteria:<br><br>Does the publication cover controlling or eliminating symptoms, |

| | |
|---|---|
| | preventing recurrence of the condition, and addressing the condition both short-term and long-term? If yes, the score should be high.<br>Provide your score (1, 2, 3, 4 or 5) and explain your reasoning. Include specific examples from the publication that support your score, or note any missing content that influenced your decision.<br>Output the final answer in json:<br>Score:[numberic],Explanation:[Words] |
| **Q11** | Q11. Does the publication describe the risks of each treatment?<br><br>Please evaluate this aspect of the publication's reliability using a 1-5 scoring system, where:<br>1 = Strongly diagree<br>2 = Diagree<br>3 = Neutral<br>4 = Agree<br>5 = Strongly agree<br><br>When assessing, consider the following criteria:<br><br>Does the publication describe the side effects, complications, and adverse reactions to treatment, both short-term and long-term? If yes, the score should be high.<br>Provide your score (1, 2, 3, 4 or 5) and explain your reasoning. Include specific examples from the publication that support your score, or note any missing content that influenced your decision.<br>Output the final answer in json:<br>Score:[numberic],Explanation:[Words] |
| **Q12** | Q12. Does the publication describe what would happen if no treatment is used?<br><br>Please evaluate this aspect of the publication's reliability using a 1-5 scoring system, where:<br>1 = Strongly diagree<br>2 = Diagree<br>3 = Neutral<br>4 = Agree<br>5 = Strongly agree<br><br>When assessing, consider the following criteria:<br><br>Does the publication describe the risks and benefits of postponing treatment, engaging in watchful waiting (monitoring the condition's progression without treatment), or permanently forgoing treatment? If yes, the score should be high.<br>Provide your score (1, 2, 3, 4 or 5) and explain your reasoning. Include specific examples from the publication that support your score, or note any missing content that influenced your decision.<br>Output the final answer in json:<br>Score:[numberic],Explanation:[Words] |

| | |
|---|---|
| **Q13** | Q13. Does the publication describe how the treatment choices affect overall quality of life?<br><br>Please evaluate this aspect of the publication's reliability using a 1-5 scoring system, where:<br>1 = Strongly diagree<br>2 = Diagree<br>3 = Neutral<br>4 = Agree<br>5 = Strongly agree<br><br>When assessing, consider the following criteria:<br><br>Does the publication describe the effects of treatment choices on day-to-day activities and the impact on relationships with family, friends, and caregivers? If yes, the score should be high.<br>Provide your score (1, 2, 3, 4 or 5) and explain your reasoning. Include specific examples from the publication that support your score, or note any missing content that influenced your decision.<br>Output the final answer in json:<br>Score:[numberic],Explanation:[Words] |
| **Q14** | Q14. Is it clear that there may be more than one possible treatment choice?<br><br>Please evaluate this aspect of the publication's reliability using a 1-5 scoring system, where:<br>1 = Strongly diagree<br>2 = Diagree<br>3 = Neutral<br>4 = Agree<br>5 = Strongly agree<br><br>When assessing, consider the following criteria:<br><br>Does the publication describe who is most likely to benefit from each treatment choice mentioned and under what circumstances, as well as suggest alternatives to consider or investigate further (including choices not fully described in the publication) before deciding whether to select or reject a particular treatment choice? If yes, the score should be high.<br>Provide your score (1, 2, 3, 4 or 5) and explain your reasoning. Include specific examples from the publication that support your score, or note any missing content that influenced your decision.<br>Output the final answer in json:<br>Score:[numberic],Explanation:[Words] |
| **Q15** | Q15. Does it provide support for shared decision-making?<br><br>Please evaluate this aspect of the publication's reliability using a 1-5 scoring system, where:<br>1 = Strongly diagree |

| | |
|---|---|
| | 2 = Diagree<br>3 = Neutral<br>4 = Agree<br>5 = Strongly agree<br><br>When assessing, consider the following criteria:<br><br>Does the publication suggest topics to discuss with family, friends, doctors, or other health professionals regarding treatment choices? If yes, the score should be high.<br>Provide your score (1, 2, 3, 4 or 5) and explain your reasoning. Include specific examples from the publication that support your score, or note any missing content that influenced your decision.<br>Output the final answer in json:<br>Score:[numberic],Explanation:[Words] |
| **Q16** | Q16. Based on the answers to all of the above questions, rate the overall quality of the publication as a source of information about treatment choices:<br><br>Please evaluate this aspect of the publication's reliability using a 1-5 scoring system, where:<br>1 = Strongly diagree<br>2 = Diagree<br>3 = Neutral<br>4 = Agree<br>5 = Strongly agree<br><br>When assessing, consider the following criteria:<br><br>A score of '1' for the publication indicates that it has serious or extensive shortcomings.<br>A score of '2' for the publication indicates that it might be useful but has some serious shortcomings.<br>A score of '3' for the publication indicates that it is potentially important but has some non-serious shortcomings.<br>A score of '4' for the publication indicates that it is useful to address the issue but has some acceptable shortcomings.<br>A score of '5' for the publication indicates that it has minimal shortcomings.<br>Provide your score (1, 2, 3, 4 or 5) and explain your reasoning. Include specific examples from the publication that support your score, or note any missing content that influenced your decision.<br>Output the final answer in json:<br>Score:[numberic],Explanation:[Words] |

Table S8 DISCERN Score Statistics by Prompt Type and Model Used in LLM Rating Process

| Response Group | | Mean | Standard Deviation | Range |
|---|---|---|---|---|
| Prompt | Model | | | |

| | | | | |
|---|---|---|---|---|
| **No Prompt** | GPT-4 | 55.82114 | 5.156617 | 43 to 67 |
| **No Prompt** | GPT-4o | 45.74797 | 5.631576 | 32 to 69 |
| **No Prompt** | Claude | 48.5935 | 5.592497 | 33 to 58 |
| **No Prompt** | GPT-3.5 | 46.46341 | 6.172776 | 32 to 57 |
| **Identity Prompt** | GPT-4 | 47.35772 | 5.232512 | 33 to 58 |
| **Identity Prompt** | GPT-4o | 46.31707 | 4.059631 | 31 to 55 |
| **Identity Prompt** | Claude | 47.73984 | 5.153126 | 33 to 58 |
| **Identity Prompt** | GPT-3.5 | 46.97561 | 5.174755 | 36 to 57 |
| **CoT-Prompt** | GPT-4 | 46.60976 | 6.185168 | 31 to 58 |
| **CoT-Prompt** | GPT-4o | 45.39837 | 5.561489 | 31 to 56 |
| **CoT-Prompt** | Claude | 48.52033 | 8.80513 | 16 to 65 |
| **CoT-Prompt** | GPT-3.5 | 46.5935 | 6.196029 | 32 to 57 |

Table S9 Tukey's Test Results of DISCERN Scores

| Between-Groups | Difference | Lower Bound | Upper Bound | P value |
|---|---|---|---|---|
| **Knee OA-Hip OA** | 0.42 | -1.03 | 1.87 | 0.94 |
| **Low Back Pain-Hip OA** | -1.07 | -2.54 | 0.39 | 0.27 |
| **Neck Pain-Hip OA** | -0.50 | -1.91 | 0.91 | 0.87 |
| **Shoulder Pain-Hip OA** | -1.14 | -2.57 | 0.30 | 0.19 |
| **Shoulder Pain-Knee OA** | -1.56 | -2.99 | -0.12 | 0.03 |
| **Low Back Pain-Knee OA** | -1.49 | -2.96 | -0.02 | 0.04 |
| **Neck Pain-Knee OA** | -0.92 | -2.33 | 0.49 | 0.39 |
| **Neck Pain-Low Back Pain** | 0.57 | -0.85 | 2.00 | 0.81 |
| **Shoulder Pain-Low Back Pain** | -0.07 | -1.52 | 1.39 | 0.99 |
| **Shoulder Pain-Neck Pain** | -0.64 | -2.03 | 0.76 | 0.72 |
| **Scenario 2-1** | 5.60 | 4.64 | 6.55 | <0.001 |
| **Scenario 3-1** | 6.50 | 5.74 | 7.26 | <0.001 |
| **Scenario 3-2** | 0.90 | -0.06 | 1.86 | 0.07 |
| **GPT-3.5-Claude** | -1.61 | -2.78 | -0.43 | 0.002 |
| **GPT-4-Claude** | 1.64 | 0.47 | 2.82 | <0.001 |
| **GPT-4o-Claude** | -2.46 | -3.64 | -1.29 | <0.001 |
| **GPT-4-GPT-3.5** | 3.25 | 2.08 | 4.43 | <0.001 |
| **GPT-4o-GPT-4** | -4.11 | -5.28 | -2.93 | <0.001 |
| **Identity Prompt- CoT Prompt** | 0.32 | -0.63 | 1.26 | 0.71 |
| **No Prompt-CoT Prompt** | 2.38 | 1.43 | 3.32 | <0.001 |
| **No Prompt-Identity Prompt** | 2.06 | 1.12 | 3.00 | <0.001 |

Table S10 Linear mixed-effects model analysis of LLM-generated response scores

Fixed effects estimates

| Variable | β (SE) | t value | P value |
|---|---|---|---|
| **Model (reference: Claude)** | | | |

| | | | |
|---|---|---|---|
| GPT-3.5 | -1.61 (0.36) | -4.53 | <0.001 |
| GPT-4 | 1.65 (0.36) | 4.64 | <0.001 |
| GPT-4o | -2.46 (0.36) | -6.94 | <0.001 |
| **Prompt (reference: CoT Prompt)** | | | |
| Identity Prompt | 0.32 (0.41) | 0.77 | 0.44 |
| No Prompt | 2.38 (0.41) | 5.78 | <0.001 |
| **Scenario (reference: Scenario 1)** | | | |
| Scenario 2 | 5.60 (0.47) | 11.94 | <0.001 |
| Scenario 3 | 6.50 (0.37) | 17.47 | <0.001 |

Overall effects of fixed factors

| Effect | F value | df | P value |
|---|---|---|---|
| **Model** | 52.34 | 3, 1107 | <0.001 |
| **Prompt** | 19.72 | 2, 369 | <0.001 |
| **Scenario** | 167.47 | 2, 369 | <0.001 |

Random effects

| Random effect | Variance | SD |
|---|---|---|
| **Question** | 4.57 | 2.14 |
| **Residual** | 23.23 | 4.82 |

Table S11 Tukey's Test Results of Readability Scores

| Readability Tool | Between-Groups | Difference | Lower Bound | Upper Bound | P value |
|---|---|---|---|---|---|
| **FRES** | Scenario 2-1 | -23.68 | -26.10 | -21.26 | <0.001 |
| | Scenario 3-1 | -21.31 | -23.23 | -19.39 | <0.001 |
| | Scenario 3-2 | 2.37 | -0.05 | 4.79 | 0.06 |
| **FKGL** | Scenario 2-1 | 3.00 | 2.57 | 3.42 | <0.001 |
| | Scenario 3-1 | 2.52 | 2.18 | 2.86 | <0.001 |
| | Scenario 3-2 | -0.48 | -0.90 | -0.05 | 0.02 |
| **GF** | Scenario 2-1 | 2.42 | 1.93 | 2.90 | <0.001 |
| | Scenario 3-1 | 1.55 | 1.17 | 1.94 | <0.001 |
| | Scenario 3-2 | -0.87 | -1.35 | -0.38 | <0.001 |
| **ARI** | Scenario 2-1 | 3.54 | 3.09 | 3.99 | <0.001 |
| | Scenario 3-1 | 2.69 | 2.34 | 3.05 | <0.001 |
| | Scenario 3-2 | -0.85 | -1.30 | -0.40 | <0.001 |
| **CLI** | Scenario 2-1 | 4.63 | 4.18 | 5.09 | <0.001 |
| | Scenario 3-1 | 3.67 | 3.31 | 4.03 | <0.001 |
| | Scenario 3-2 | -0.96 | -1.42 | -0.51 | <0.001 |
| **SMOG** | Scenario 2-1 | 1.75 | 1.41 | 2.10 | <0.001 |
| | Scenario 3-1 | 1.39 | 1.12 | 1.67 | <0.001 |
| | Scenario 3-2 | -0.36 | -0.71 | -0.01 | 0.04 |
| **FRES** | GPT-3.5-Claude | 0.10 | -3.28 | 3.49 | 0.99 |
| | GPT-4-Claude | -0.56 | -3.94 | 2.83 | 0.97 |
| | GPT-4o-Claude | 0.57 | -2.82 | 3.95 | 0.97 |
| | GPT-4-GPT-3.5 | -0.66 | -4.05 | 2.72 | 0.96 |
| | GPT-4o-GPT-3.5 | 0.46 | -2.92 | 3.85 | 0.98 |
| | GPT-4o-GPT-4 | 1.13 | -2.26 | 4.51 | 0.83 |
| **FKGL** | GPT-3.5-Claude | -0.43 | -0.96 | 0.10 | 0.16 |

| | | | | | |
|---|---|---|---|---|---|
| | GPT-4-Claude | 0.08 | -0.45 | 0.61 | 0.98 |
| | GPT-4o-Claude | -0.64 | -1.17 | -0.11 | 0.01 |
| | GPT-4-GPT-3.5 | 0.51 | -0.02 | 1.04 | 0.07 |
| | GPT-4o-GPT-3.5 | -0.21 | -0.74 | 0.32 | 0.75 |
| | GPT-4o-GPT-4 | -0.72 | -1.25 | -0.18 | <0.001 |
| **GF** | GPT-3.5-Claude | -0.94 | -1.49 | -0.39 | <0.001 |
| | GPT-4-Claude | -0.12 | -0.67 | 0.43 | 0.94 |
| | GPT-4o-Claude | -1.84 | -2.39 | -1.29 | <0.001 |
| | GPT-4-GPT-3.5 | 0.82 | 0.27 | 1.37 | <0.001 |
| | GPT-4o-GPT-3.5 | -0.90 | -1.46 | -0.35 | <0.001 |
| | GPT-4o-GPT-4 | -1.72 | -2.27 | -1.17 | <0.001 |
| **ARI** | GPT-3.5-Claude | -0.50 | -1.07 | 0.07 | 0.11 |
| | GPT-4-Claude | 0.01 | -0.56 | 0.58 | 0.99 |
| | GPT-4o-Claude | -0.70 | -1.27 | -0.12 | 0.01 |
| | GPT-4-GPT-3.5 | 0.51 | -0.06 | 1.08 | 0.10 |
| | GPT-4o-GPT-3.5 | -0.19 | -0.76 | 0.38 | 0.82 |
| | GPT-4o-GPT-4 | -0.70 | -1.27 | -0.13 | 0.01 |
| **CLI** | GPT-3.5-Claude | -0.39 | -1.02 | 0.23 | 0.37 |
| | GPT-4-Claude | -0.15 | -0.78 | 0.48 | 0.93 |
| | GPT-4o-Claude | -0.78 | -1.41 | -0.16 | 0.01 |
| | GPT-4-GPT-3.5 | 0.24 | -0.39 | 0.87 | 0.75 |
| | GPT-4o-GPT-3.5 | -0.39 | -1.02 | 0.24 | 0.38 |
| | GPT-4o-GPT-4 | -0.63 | -1.26 | 0.00 | 0.05 |
| **SMOG** | GPT-3.5-Claude | -0.65 | -1.05 | -0.24 | <0.001 |
| | GPT-4-Claude | 0.02 | -0.38 | 0.42 | 0.99 |
| | GPT-4o-Claude | -0.93 | -1.33 | -0.53 | <0.001 |
| | GPT-4-GPT-3.5 | 0.67 | 0.27 | 1.07 | <0.001 |
| | GPT-4o-GPT-3.5 | -0.28 | -0.69 | 0.12 | 0.27 |
| | GPT-4o-GPT-4 | -0.95 | -1.35 | -0.55 | <0.001 |
| **FRES** | Identity Prompt-CoT Prompt | -2.99 | -5.66 | -0.32 | 0.02 |
| | No Prompt-CoT Prompt | -1.55 | -4.22 | 1.12 | 0.36 |
| | No Prompt-Identity Prompt | 1.44 | -1.23 | 4.10 | 0.42 |
| **FKGL** | Identity Prompt-CoT Prompt | 0.35 | -0.07 | 0.77 | 0.12 |
| | No Prompt-CoT Prompt | 0.44 | 0.02 | 0.86 | 0.04 |
| | No Prompt-Identity Prompt | 0.09 | -0.33 | 0.51 | 0.87 |
| **GF** | Identity Prompt-CoT Prompt | 0.02 | -0.43 | 0.46 | 0.99 |
| | No Prompt-CoT Prompt | 0.56 | 0.12 | 1.01 | 0.01 |
| | No Prompt-Identity Prompt | 0.55 | 0.10 | 0.99 | 0.01 |
| **ARI** | Identity Prompt-CoT Prompt | 0.43 | -0.03 | 0.88 | 0.07 |
| | No Prompt-CoT Prompt | 0.44 | -0.01 | 0.89 | 0.06 |
| | No Prompt-Identity Prompt | 0.01 | -0.44 | 0.46 | 0.99 |
| **CLI** | Identity Prompt-CoT Prompt | 0.69 | 0.19 | 1.19 | <0.001 |
| | No Prompt-CoT Prompt | 0.39 | -0.11 | 0.88 | 0.16 |

| | | | | | |
|---|---|---|---|---|---|
| | No Prompt-Identity Prompt | -0.30 | -0.80 | 0.19 | 0.32 |
| **SMOG** | Identity Prompt-CoT Prompt | 0.18 | -0.14 | 0.51 | 0.37 |
| | No Prompt-CoT Prompt | 0.47 | 0.15 | 0.79 | <0.001 |
| | No Prompt-Identity Prompt | 0.29 | -0.04 | 0.61 | 0.09 |
| **FRES** | Knee OA-Hip OA | 0.02 | -4.02 | 4.05 | 0.99 |
| | Low Back Pain-Hip OA | 5.45 | 1.37 | 9.53 | <0.001 |
| | Neck Pain-Hip OA | 1.01 | -2.91 | 4.93 | 0.96 |
| | Shoulder Pain-Hip OA | -1.71 | -5.70 | 2.29 | 0.77 |
| | Low Back Pain-Knee OA | 5.43 | 1.35 | 9.51 | <0.001 |
| | Neck Pain-Knee OA | 0.99 | -2.93 | 4.91 | 0.96 |
| | Shoulder Pain-Knee OA | -1.73 | -5.72 | 2.27 | 0.76 |
| | Neck Pain-Low Back Pain | -4.44 | -8.41 | -0.47 | 0.02 |
| | Shoulder Pain-Low Back Pain | -7.15 | -11.19 | -3.12 | <0.001 |
| | Shoulder Pain-Neck Pain | -2.71 | -6.60 | 1.17 | 0.31 |
| **FKGL** | Knee OA-Hip OA | 0.07 | -0.57 | 0.71 | 0.99 |
| | Low Back Pain-Hip OA | -0.73 | -1.37 | -0.08 | 0.02 |
| | Neck Pain-Hip OA | -0.10 | -0.72 | 0.52 | 0.99 |
| | Shoulder Pain-Hip OA | 0.25 | -0.38 | 0.88 | 0.82 |
| | Low Back Pain-Knee OA | -0.80 | -1.44 | -0.15 | 0.01 |
| | Neck Pain-Knee OA | -0.17 | -0.79 | 0.45 | 0.94 |
| | Shoulder Pain-Knee OA | 0.18 | -0.45 | 0.81 | 0.94 |
| | Neck Pain-Low Back Pain | 0.62 | 0.00 | 1.25 | 0.05 |
| | Shoulder Pain-Low Back Pain | 0.98 | 0.34 | 1.62 | <0.001 |
| | Shoulder Pain-Neck Pain | 0.35 | -0.26 | 0.97 | 0.52 |
| **GF** | Knee OA-Hip OA | 0.11 | -0.57 | 0.79 | 0.99 |
| | Low Back Pain-Hip OA | -0.62 | -1.31 | 0.07 | 0.10 |
| | Neck Pain-Hip OA | 0.24 | -0.42 | 0.91 | 0.85 |
| | Shoulder Pain-Hip OA | -0.01 | -0.69 | 0.66 | 0.99 |
| | Low Back Pain-Knee OA | -0.73 | -1.42 | -0.04 | 0.03 |
| | Neck Pain-Knee OA | 0.14 | -0.52 | 0.80 | 0.98 |
| | Shoulder Pain-Knee OA | -0.12 | -0.79 | 0.55 | 0.99 |
| | Neck Pain-Low Back Pain | 0.86 | 0.20 | 1.53 | <0.001 |
| | Shoulder Pain-Low Back Pain | 0.61 | -0.07 | 1.29 | 0.11 |
| | Shoulder Pain-Neck Pain | -0.26 | -0.91 | 0.40 | 0.82 |
| **ARI** | Knee OA-Hip OA | 0.35 | -0.33 | 1.04 | 0.62 |
| | Low Back Pain-Hip OA | -0.64 | -1.33 | 0.05 | 0.09 |
| | Neck Pain-Hip OA | -0.13 | -0.79 | 0.54 | 0.99 |
| | Shoulder Pain-Hip OA | 0.35 | -0.33 | 1.03 | 0.62 |
| | Low Back Pain-Knee OA | -1.00 | -1.69 | -0.30 | <0.001 |
| | Neck Pain-Knee OA | -0.48 | -1.15 | 0.18 | 0.28 |
| | Shoulder Pain-Knee OA | 0.00 | -0.68 | 0.67 | 0.99 |
| | Neck Pain-Low Back Pain | 0.51 | -0.16 | 1.19 | 0.23 |
| | Shoulder Pain-Low Back Pain | 0.99 | 0.31 | 1.68 | <0.001 |

| | | | | | |
|---|---|---|---|---|---|
| | Shoulder Pain-Neck Pain | 0.48 | -0.18 | 1.14 | 0.28 |
| **CLI** | Knee OA-Hip OA | 0.40 | -0.35 | 1.15 | 0.59 |
| | Low Back Pain-Hip OA | -0.70 | -1.46 | 0.07 | 0.09 |
| | Neck Pain-Hip OA | -0.10 | -0.83 | 0.63 | 0.99 |
| | Shoulder Pain-Hip OA | 0.50 | -0.24 | 1.25 | 0.35 |
| | Low Back Pain-Knee OA | -1.10 | -1.86 | -0.33 | <0.001 |
| | Neck Pain-Knee OA | -0.50 | -1.23 | 0.23 | 0.34 |
| | Shoulder Pain-Knee OA | 0.10 | -0.64 | 0.85 | 0.99 |
| | Neck Pain-Low Back Pain | 0.60 | -0.14 | 1.34 | 0.18 |
| | Shoulder Pain-Low Back Pain | 1.20 | 0.44 | 1.95 | <0.001 |
| | Shoulder Pain-Neck Pain | 0.60 | -0.12 | 1.32 | 0.16 |
| **SMOG** | Knee OA-Hip OA | 0.10 | -0.39 | 0.59 | 0.98 |
| | Low Back Pain-Hip OA | -0.50 | -1.00 | 0.00 | 0.05 |
| | Neck Pain-Hip OA | -0.04 | -0.52 | 0.44 | 0.99 |
| | Shoulder Pain-Hip OA | 0.08 | -0.40 | 0.57 | 0.99 |
| | Low Back Pain-Knee OA | -0.60 | -1.10 | -0.11 | 0.01 |
| | Neck Pain-Knee OA | -0.14 | -0.62 | 0.33 | 0.92 |
| | Shoulder Pain-Knee OA | -0.02 | -0.51 | 0.46 | 0.99 |
| | Neck Pain-Low Back Pain | 0.46 | -0.02 | 0.94 | 0.07 |
| | Shoulder Pain-Low Back Pain | 0.58 | 0.09 | 1.07 | 0.01 |
| | Shoulder Pain-Neck Pain | 0.12 | -0.35 | 0.59 | 0.96 |
