# Supplementary material for: Evaluation of Large Language Models in Generating Physical Exercise Rehabilitation Programs for Musculoskeletal Disorders Across Multiple Clinical Scenarios
Source: Healthcare (Basel). 2026 Aug 4;14(15):2389. doi: 10.3390/healthcare14152389 (PMC13465567; doi:10.3390/healthcare14152389)
Supplement: Supplementary file 1 [file healthcare-14-02389-s001.zip › Supporting Information S2.pdf]

Supporting Information S2

1. Strategy of random sampling

1.1 Calculating the necessary sample size

After LLM's rating process, we recorded 23616 scores for 1476 responses generated by LLMs (16 DISCERN scores for each rehabilitation programs). We calculated the necessary sample size by using the formula in Table 1 under a 95% confidence level and 2% margin of error. To ensure a sample size that represents the population, we set the standard deviation at 0.5 and we assumed that the sampling distribution is normally distributed.

Table S12. Formula for Calculating the Size of Random Sampling and Z-scores Distribution

Formula for Calculating the Size of Random Sampling

$$\text{Necessary Sample Size} = \frac{\text{Z\_scores}^2 \times StdDev \times (1 - StdDev)}{(margin\ of\ error)^2}$$

***StdDev= Standard Deviation**

Z-scores under normally distribution

Confidence Level	Z-score
**0.70**	1.04
**0.75**	1.15
**0.80**	1.28
**0.85**	1.44
**0.92**	1.75
**0.95**	1.96
**0.96**	2.05
**0.98**	2.33
**0.99**	2.58
**0.999**	3.29
**0.9999**	3.89
**0.99999**	4.42

1.2 Sampling randomly

We give an ID number to each DISCERN score with its explanation from 1 to 23616 and using R software (version 3.6.0) to generate 2180 pseudorandom number also from 1 to 23616 (true random numbers are based on physical phenomena like atmospheric noise and other quantum phenomena). We matched the ID number of the DISCERN scores and the random numbers to determine the sample for further experts' validation.

2. Strategy of experts' validation of LLM's evaluation

We asked one doctor and one therapist to evaluate quality of the LLM's evaluation scores by using a five-point Likert scale (1= very poor quality, 2= poor quality, 3= moderate quality, 4= good quality and 5= excellent quality). We asked them to rate the quality according to the tips in Table 2.

Table S13. Tips for experts' validation of LLM's evaluation

Tips for experts' validation of LLM's evaluation

Score	Tips
**1**	LLM's score is completely unreasonable, completely deviated to DISCERN question's requirement.<br>The explanation is full of ambiguous description.
**2**	LLM's score is unreasonable, deviated to DISCERN question's requirement.<br>The explanation is clear but cannot support the DISCERN score.
**3**	LLM's score is reasonable, conform to DISCERN question's requirement.<br>But the explanation is not clear and cannot support the DISCERN score.
**4**	LLM's score is reasonable, conform to DISCERN question's requirement.<br>The explanation is not clear enough but can support the DISCERN score.
**5**	LLM's score is reasonable, conform to DISCERN question's requirement.<br>The explanation is clear without any vague description and can support the DISCERN score.
